# Application of advanced biomaterials in photothermal therapy for malignant bone tumors

**DOI:** 10.1186/s40824-023-00453-z

**Published:** 2023-11-15

**Authors:** Bo Chao, Jianhang Jiao, Lili Yang, Yang Wang, Weibo Jiang, Tong Yu, Linfeng Wang, He Liu, Han Zhang, Zhonghan Wang, Minfei Wu

**Affiliations:** Department of Orthopedics, The Second Hospital of Jilin University, Changchun, 130041 People’s Republic of China

**Keywords:** Malignant bone tumors, Photothermal therapy, Hyperthermia, Advanced biomaterials, Combination therapy

## Abstract

Malignant bone tumors are characterized by severe disability rate, mortality rate, and heavy recurrence rate owing to the complex pathogenesis and insidious disease progression, which seriously affect the terminal quality of patients' lives. Photothermal therapy (PTT) has emerged as an attractive adjunctive treatment offering prominent hyperthermal therapeutic effects to enhance the effectiveness of surgical treatment and avoid recurrence. Simultaneously, various advanced biomaterials with photothermal capacity are currently created to address malignant bone tumors, performing distinctive biological functions, including nanomaterials, bioceramics (BC), polymers, and hydrogels et al. Furthermore, PTT-related combination therapeutic strategies can provide more significant curative benefits by reducing drug toxicity, improving tumor-killing efficiency, stimulating anti-cancer immunity, and improving immune sensitivity relative to monotherapy, even in complex tumor microenvironments (TME). This review summarizes the current advanced biomaterials applicable in PTT and relevant combination therapies on malignant bone tumors for the first time. The multiple choices of advanced biomaterials, treatment methods, and new prospects for future research in treating malignant bone tumors with PTT are generalized to provide guidance.

**Graphical Abstract:**

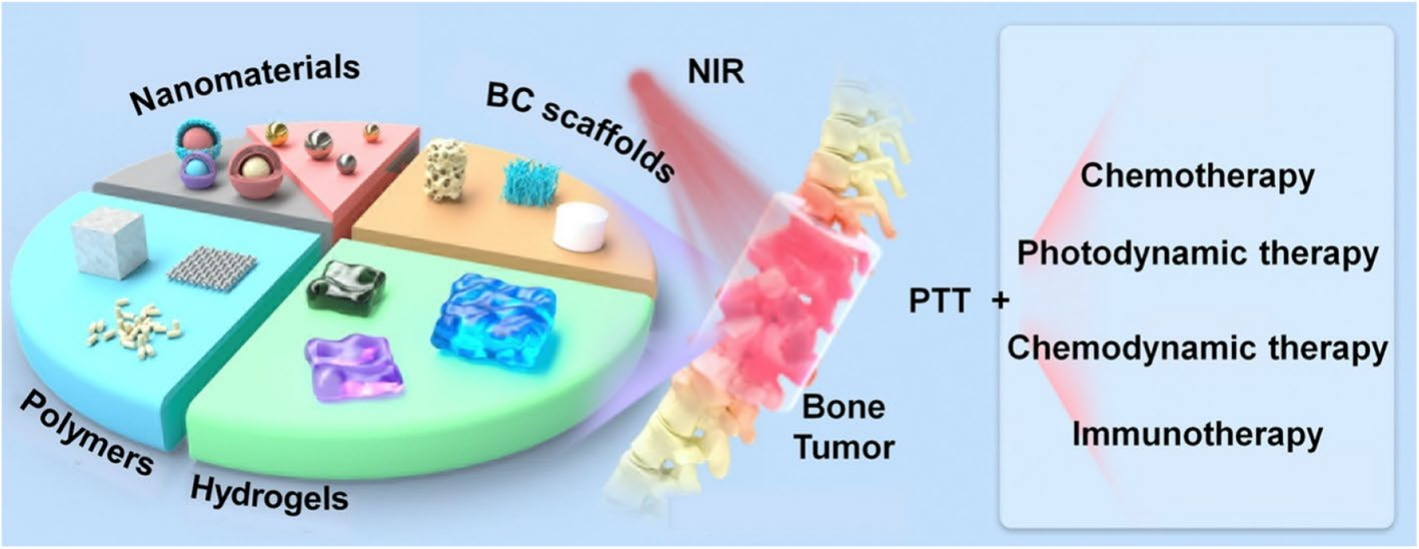

Malignant bone tumors seriously affect the terminal quality of patients' lives. Photothermal therapy (PTT) has emerged as an attractive adjunctive treatment enhancing the effectiveness of surgical treatment and avoiding recurrence. In this review, advanced biomaterials applicable in the PTT of malignant bone tumors and their distinctive biological functions are comprehensively summarized for the first time. Simultaneously, multiple PTT-related combination therapeutic strategies are classified to optimize practical clinical issues, contributing to the selection of biomaterials, therapeutic alternatives, and research perspectives for the adjuvant treatment of malignant bone tumors with PTT in the future.

## Introduction

Malignant bone tumors are characterized by a severe disability rate, mortality rate, and heavy recurrence rate that seriously affect the terminal quality of patients' lives [1]. According to the tissue origin, malignant bone tumors can be divided into primary and metastatic tumors. Primary malignant bone tumors mainly include osteosarcoma, Ewing sarcoma, and chondrosarcoma [2]. Osteosarcoma is the most common primary bone tumor and the third-most common cancer in minors. The risk factors of osteosarcoma comprise the patient's treatment history of radiotherapy or chemotherapy, Paget and hereditary retinoblastoma, and other genetic diseases. However, its definitive etiology is still obscure [3]. Metastatic bone tumors are defined as secondary tumor that migrates from other parts of the body and colonizes the bone, occurring in 65–80% patients with terminal breast or prostate cancer and in 35–42% patients with terminal thyroid, lung, or kidney cancer, and associated with a higher incidence rate than primary bone tumors [4]. Owing to the specific microenvironment of bone tissue, migrating tumor cells prefer to be recruited from the vasculature. In addition, tumor cells can also interfere with the metabolic balance of bone tissue, leading to osteolytic or osteogenic destruction in the metastatic lesion [5, 6]. Both primary and metastatic bone tumors attribute to devastating outcomes such as pain, hypercalcemia, nerve compression syndromes, and pathological fractures [7].

Notably, the current treatment of malignant bone tumors focuses on the curettage of the tumor and subsequently reconstruction of bone integrity. However, the overall physical condition of patients is extremely weak in the terminal stage, and extensive resection surgery brings great burdens to patients, and the recurrence rate is still ranging between 2 and 17% [8, 9]. Moreover, to minimize recurrence, bone tissue surrounding the tumor is resected as much as possible, causing critical tumor-derived bone defects in the surgical area subsequently [10]. Appropriately, biomaterials of bone tissue engineering can be instrumental in this process, as biomaterials inserted into tumor-derived bone defects not only restore bone stability but also remove residual tumor cells from the margins in combination with other therapeutics [5]. Although the overall survival and limb salvage rates have both improved, efficacy remains to be optimized and systemic side effects cannot be ignored [11, 12]. Therefore, a more efficient, safe, and versatile treatment strategy for bone tumors desperately deserves consideration.

PTT, a novel adjuvant tumor therapeutic strategy that converts light energy into heat energy, subjects tumor cells to high temperatures between 41 °C and 45 °C thereby killing them [13]. The high temperature increases vascular permeability and blood flow to the tissue simultaneously, alleviating hypoxia of tumors, thus expeditiously improving the curative effect and enrichment capacity of anticancer drugs [14]. Exogenous stimuli are indispensable in this process, especially near-infrared laser (NIR). Laser wavelengths in the NIR range (NIR, 650–1700 nm) provide deeper tissue penetration due to less scattering and reabsorption, lower energy, and in comparison to visible light (< 1 mm) [15]. Furthermore, with the relatively high absorption coefficients of human tissues in the visible range of the electromagnetic spectrum, the excitation wavelengths applied in PTT are generally specified in the "biological window", the NIR-I (650–950 nm) or NIR-II spectral ranges (1000–1700 nm) [16]. Notably, for protection against potential skin damage during PTT, the maximum permissible exposure (MPE) for the 808 nm laser is limited to 0.33 W/cm^2^ [17]. Whereas the low energy of the long wavelength photons will allow for a corresponding enhancement of the MPE, thereby allowing NIR-II with a relatively high MPE [18]. Therefore, long-wavelength NIR promises better prospects for clinical applications.

Indeed, photosensitizers are instrumental in this process, being responsible for converting light into heat energy under NIR radiation [19, 20]. Significantly, the therapeutic efficacy depends on diverse parameters of the photosensitizer, including size effect, photothermal conversion efficiency, surface chemistry, physiological stability, and biodegradability [21]. Classical photosensitizers generally consist of indocyanine green (ICG) [22], gold (Au) [23, 24], Fe_3_O_4_ [25, 26], carbon [27, 28], graphene oxide (GO) [29, 30], and black phosphorus (BP) [31] et al.; however, the poor biostability, short blood half-life, and limited accumulation at the tumor site observed with some conventional photosensitizers like ICG, which is affecting their therapeutic efficacy seriously [32]. Consequently, current research has discovered innovative strategies to adjust the size, surface functionalization, and coating of photosensitizers, even loading ICG by nanomaterials to possess efficient precision PTT [33].

Meantime, recent research reveals that the strategy of combining biomaterials with PTT exclusively for bone tumor treatment exhibits preferable capabilities to eliminate tumor tissue and reconstruct bone tissue without significant side effects [34]. Following the biological, chemical, and physical criteria for multifunctional photothermal biomaterials, photosensitizers are frequently employed as nanomaterials for modification or components incorporated into biomaterials of bone tissue engineering, including nanomaterials, bioceramic (BC) scaffolds, hydrogels, and polyether ether ketone (PEEK) et al. [35–37]. Research on multiple photothermal materials is actually an attempt to identify biomaterials more applicable for the treatment of bone tumors, not limited to being minimally invasive, non-toxic, and highly effective, but also regenerating bone to repair tumor-derived bone defects [38].

However, the application of PTT in deep and internal bone tumors is severely compromised as a result of thermal resistance, limited irradiation area, and depth of penetration. Normal tissue necrosis and pro-inflammatory response may be produced by exorbitant PTT if the power density of NIR is solely increased, by which immune surveillance and immune editing were inhibited subsequently to weaken the immune response [39]. Therefore, combination therapy with PTT has emerged as a prospective alternative to monotherapy for the convenience of promoting therapeutic performance [40, 41]. PTT presents a promising potential for combination with alternative treatments due to its low toxicity, minimal side effects, and simplicity of operation. The combination of chemotherapy, photodynamic therapy (PDT), chemodynamic therapy (CDT), and immunotherapy augments the effectiveness of oncology treatment and even the repair of bone defects [42, 43].

**Fig. 1:**
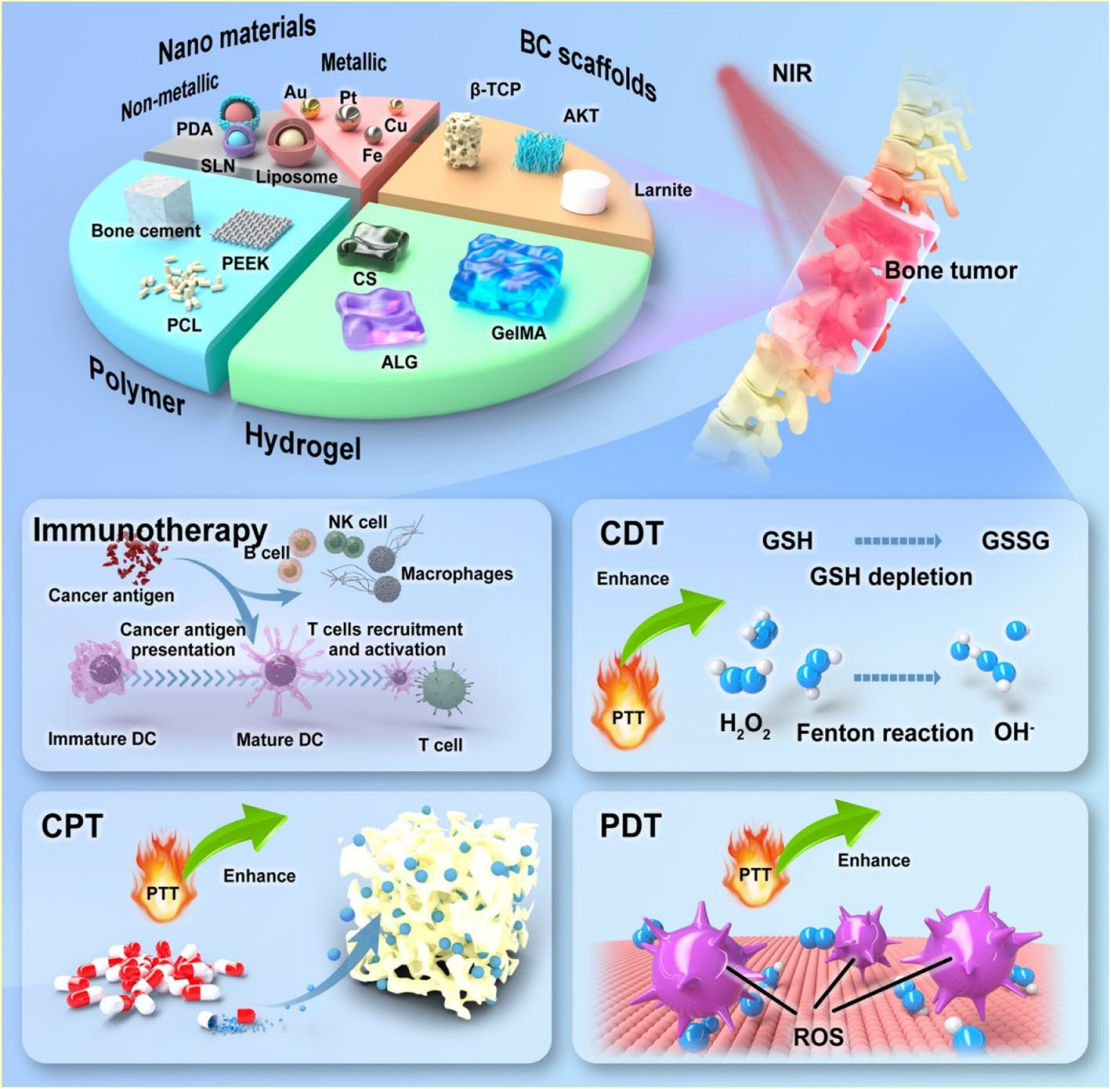
The schematic of application of advanced biomaterials and combination therapeutic strategies in PTT for malignant bone tumors

In this review, various biomaterials currently applied in the study of PTT for malignant bone tumors are systematically classified for the first time, as well as the loading modality and functions of photosensitizers are introduced to provide a reference for elevating PTT efficacy. Furthermore, we also discuss the combination of PTT with several therapeutic strategies for reducing side effects, improving autoimmunity, and mobilizing TME to depress malignant tumor cells. This review focuses on the multiple choices of available biomaterials and combination therapeutic strategies of PPT, for reference to future research in the treatment of malignant bone tumors (Fig. 1).

## Biomaterials for malignant bone tumors treated with PTT

The application of biomaterials for bone tissue engineering as fillers is indispensable since the treatment of bone tumors is frequently associated with significant tumor-derived bone defects [5]. As mentioned above, more impressive tumor suppression can be accomplished by appropriately matching the photothermal capabilities with the biomaterial properties. Moreover, as diverse as the unique advantages and applications are, different biomaterials exert their specific biological effects in the PTT of malignant bone tumors. Significantly, the photothermal properties of different biomaterials are principally based on photosensitizers, and most photosensitizers consist of nanomaterials. The reason for presenting a classification of different photothermal materials in this section, including nanomaterials, is related to the differential advantages of the physicochemical properties and characterization of specific biomaterials in the efficacy of bone tumors.

### Nanoscale biomaterials

Nanomaterials demonstrated enormous advantages in improving the therapeutic efficacy of malignant bone tumors [44], including tunable penetration ability, sustainability in the circulation system, precise targeting, enhanced drug utilization, and PTT-triggered drug release [45]. Nanomaterials are fundamentally divided into metallic and non-metallic nanomaterials, respectively combining its inherent properties with PTT will provide appreciable benefits in treating malignant bone tumors [46]. Metallic nanomaterials are the collective expression for nanomaterials with metallic properties composed of metallic elements or mainly metallic elements. Non-metallic nanomaterials, generally known as other than metallic nanomaterials, are the materials obtained from organic and inorganic materials or various organic and inorganic materials in appropriate combinations after certain physical or chemical treatments [47].

#### Metallic nanomaterials

**Fig. 2:**
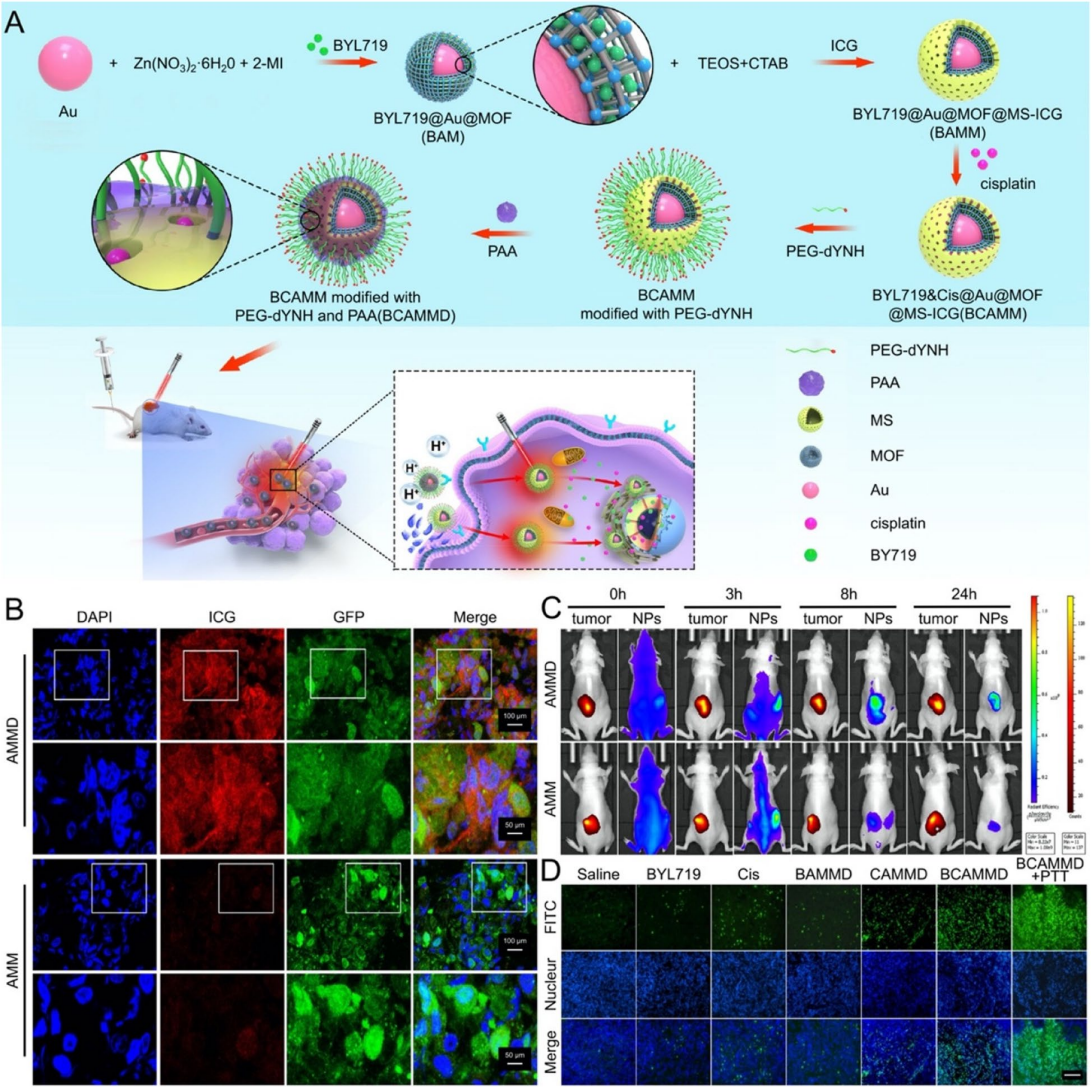
**A** Schematic illustration of the fabrication for theranostic GNP and the antitumor mechanisms in the tumor cell. **B** The fluorescence signal of NP and green fluorescent protein-labeled A549 cells in the frozen section of the tumor 48 h after administration of NP. Scale bar: 100 µm and 50 µm. **C** The bioluminescence (labeled A549 cells) and fluorescence (NP in blue) images of the mice with spinal metastasis at 0, 3, 8, and 24 h after injection. **D** TUNEL fluorescence images described apoptosis in tumor slices. Abbreviations: Alpelisib (BYL719), tetraethyl orthosilicate (TEOS), Cetyltrimethyl Ammonium Bromide (CTAB), indocyanine green (ICG), polyethylene glycol (PEG), poly acrylic acid (PAA), BYL719@Au@MOF-BAM, BYL719@Au@MOF@MS-ICG (BAMM), BYL719&Cisplatin@Au@MOF@MS-ICG (BCAMM), BCAMM modified with dYNH targeting peptide (BCAMMD). Scale bar: 20 µm.  Reproduced with permission from Ref. [55]

Metallic nanomaterials have attracted great attention in PTT for reasons of their specific physical properties, including gold-based nanomaterials, platinum-based nanomaterials, rare metal and alloy nanomaterials, and multi-element metal–organic frameworks (MOFs) [48]. The mechanism of energy absorption in PTT occurs as a result of the interaction between light and the conducting electrons on the surface of metallic nanomaterials, leading to the subsequent release of a portion of this energy as heat. When the illumination wavelength resonates with the surface plasma frequency (localized surface plasmon resonance, LSPR), absorption contributes to optimal heat dissipation [49]. Gold nanoparticles (GNPs) are the most versatile substances applied in photoresponsive hyperthermia for the reason of their excellent biocompatibility and modifiability [50]. In particular, GNPs with different sizes can absorb incident photons and convert them into heat, as well as with smaller sizes exhibit better NIR absorption and scattering due to their optimal harmonized optical resonance [51], which can be efficiently converted into heat energy to ensure effective PTT even low-radiation energy [52]. The recurrence of malignant bone tumors after surgical resection is consistently one of the major contributors to poor prognosis. GNP-aided PTT, acting as a new adjuvant therapy, can eliminate residual tumor cells on the resection edge to prevent tumor recurrence. Inspired by this, Marina et al. performed subtotal tumor resection in tumor-bearing mice to simulate the clinical situation of incomplete tumor resection [53], afterwards, administrating GNP-aided PTT in the surgical area exhibited permanent tumor suppression with a higher survival rate than without PTT, thus demonstrating the potential of GNP-aided PTT as adjuvant therapy after tumor resection. Similarly, to enhance the tumor-killing effect, various modifications of metallic nanomaterials have been suggested in the follow-up research [22, 54]. A double drug-loaded therapeutic GNP was wrapped around by a dual shell consisting of MOF and mesoporous silica [55], guaranteeing the stability of drugs and enabling pH-responsive drug delivery in the acidic TME. Additionally, the targeted peptide (dYNH) and indocyanine green (ICG) were assembled on the outermost layer to ensure the accurate targeting of tumor cells (Fig. 2A). Progressive enhancement of fluorescence signal at the tumor site was observed in vivo at different times points post-injection (Fig. 2B, C), thus significantly killing bone tumor cells (Fig. 2D).

Excellent targeting performance is an indispensable prerequisite for enhancing therapeutic efficacy [56], for the reason of NPs without targeting capability necessitate extensive amounts of nanosystems, intense laser power, and elevated PTT temperatures, which susceptible normal tissue to burns [57]. Therefore, to improve the targeting ability of NPs, cell products including tumor cell targeting ligands, bone cell biomarkers, and cell membranes were applied for surface modification of NP, the biocompatibility and bioavailability of NP are significantly improved by such a coating strategy [58, 59]. Typically, the membranes of normal human cells such as mesenchymal stem cells (MSC), macrophages, red blood cells, and platelets were extracted followed by processed onto the surface of NPs because escaping the attack of macrophages is critical for the therapeutic effects of nanomaterials [60, 61]. Kim et al. have been dedicated to coating gold nanostars with a mixture of erythrocyte membrane and platelet membrane to evade rapid clearance of macrophages [62]. It was obvious that such a double-membrane camouflage strategy not only equipped the NP with cancer-targeting ability but promoted the tumor-killing effectiveness of PTT. Alendronate sodium (ALN) is considerably preferred because of its bone-targeting properties [63], but its circulation period in vivo remains to be elevated further. Whereas ALN complexes encapsulated by hyaluronic acid are capable of overcoming physiological barriers, enhancing the accumulation of modified nanomaterials in the bone tumor region in vivo and the uptake of target cells in vitro [64].

Peptide modification is another strategy that bestows NP with tumor-targeting ability [65]. The surface of metallic NPs can be modified by antibodies such as CD133, CD163, CD271, and HER-2, which are intimately associated with tumor cell behavior such as self-renewal, differentiation potential, signal transduction, and drug tolerance [66, 67]. These antibodies can anchor tumor cells specifically, enabling NP excellent tumor-targeting ability and possessing better anti-tumor effects than those without modification [68]. For instance, GNPs accompanied by CD133-targeted peptide were employed in accurately amassing in osteosarcoma, significantly accelerating the temperature of tumor tissue than surrounding normal tissue with the intensive introduction of GNPs, killing osteosarcoma cells subsequently [69]. Necessarily effective not only when targeting tumor tissue, but considerable therapeutic benefits can also be exhibited by targeting bone fragments. A dendritic NP was modified by eight aspartic oligopeptides (Asp8) with excellent affinity to hydroxyapatite (HA), primarily orientating to the bone tissue around tumors by Asp8 and inhibiting tumor growth consequentially through PTT. During the treatment period, none of the mice in the experimental group showed any significant decrease in body weight indicating negligible systemic toxicity [70].

Apart from ordinary metallic elements, some rare metallic elements are used to synthesize NPs for treating malignant bone tumors, expanding the horizon of optional metallic NP in PTT [71, 72]. For example, a novel 150 nm oxygen-rich vacancy tungsten bronze NP (NaXWO_3_) possessed significant tumor ablation effects under NIR predicated on the increased expression of the apoptotic markers Bax and p-Akt. Additionally, the expressions of osteoclastic RANKL and Sclerostin were inhibited, disturbing downstream osteoclast genesis. The NaXWO_3_ NP provided new insights into PTT for the application of rare metallic elements [73]. Alternatively, GdPO_4_/chitosan (CS)/Fe_3_O_4_ NP could simultaneously realize bone defect healing and thermal ablation of postoperative residual tumor cells; Gd^3+^, as a rare metallic element of it, promoted the proliferation and differentiation of osteoblasts, activating the BMP-2/Smad/RUNX2 signaling pathway to facilitate bone regeneration, simultaneously inducing M2 polarization of macrophages to stabilize the vascularized system and provide oxygen and nutrition for osteogenesis [74]. NP composed of diverse rare elements therefore could achieve multifunction involving photothermal ablation of postoperative residual tumor and bone defect healing, which possesses promising prospects in treating malignant bone tumors.

Alloyed nanomaterials are also a popular domain in the PTT of bone tumors [75–77]. NiTi alloy was manufactured into a multi-scale hierarchical structure composed of a three-dimensional micro-nano structure. The alloyed nanomaterials significantly inhibited the growth of osteosarcoma and simultaneously accelerated the osteogenic differentiation of osteoblasts positively through PTT [78]. Similarly, an innovative approach to modifying NiTi was proposed, femtosecond laser, fabricating groovelike micro-nanostructures to reinforce osseointegration by intensive contact guidance. Finally, polydopamine (PDA) modification was applied to enhance the photothermal properties and chemical stability of NiTi, completely eradicating osteosarcoma in mice with desirable osteogenic effects [79].

#### Non-metallic nanomaterials

Non-metallic nanomaterials primarily include PDA NP, silica NP (SLN), graphene, liposome, and protein NP. Notably, the mechanism of photothermal conversion of non-metallic nanomaterials involves the activation of electrons within the molecules to produce electron–hole pairs when under NIR. These electron–hole pairs are transmitted consistently within the material and assimilated into thermal energy ultimately [80]. In addition, some non-metallic carbon-based nanomaterials are characterized by strong absorption of NIR, and the molecules will be excited from the ground state to the lowest excited singlet state when they are exposed to NIR. The non-radiative relaxation pathway decays back to the ground state in a manner that produces a photothermal effect subsequently, due to molecules in the excited state being unstable [81]. In contrast to metallic nanomaterials, carbon-based nanomaterials are not affected by their geometry as they exhibit significant absorption and scattering across the entire biological spectral range. And some non-metallic nanomaterials are rich in catechol/quinone moieties thereby anchoring specific molecules to biomaterials through chemical or physical bonding [82]. The excellent biocompatibility, biodegradability, and structural versatility are also among the attractions of non-metallic nanomaterials to researchers [83]. Currently, the application of NIR-responsive thermogenic polymer nanoplatforms is of increased interest in cancer therapy, principally attributed to responsive polymer NP in target tissues triggered to deliver tumor therapeutic agents when subjected to specific stimulation [84].

**Fig. 3:**
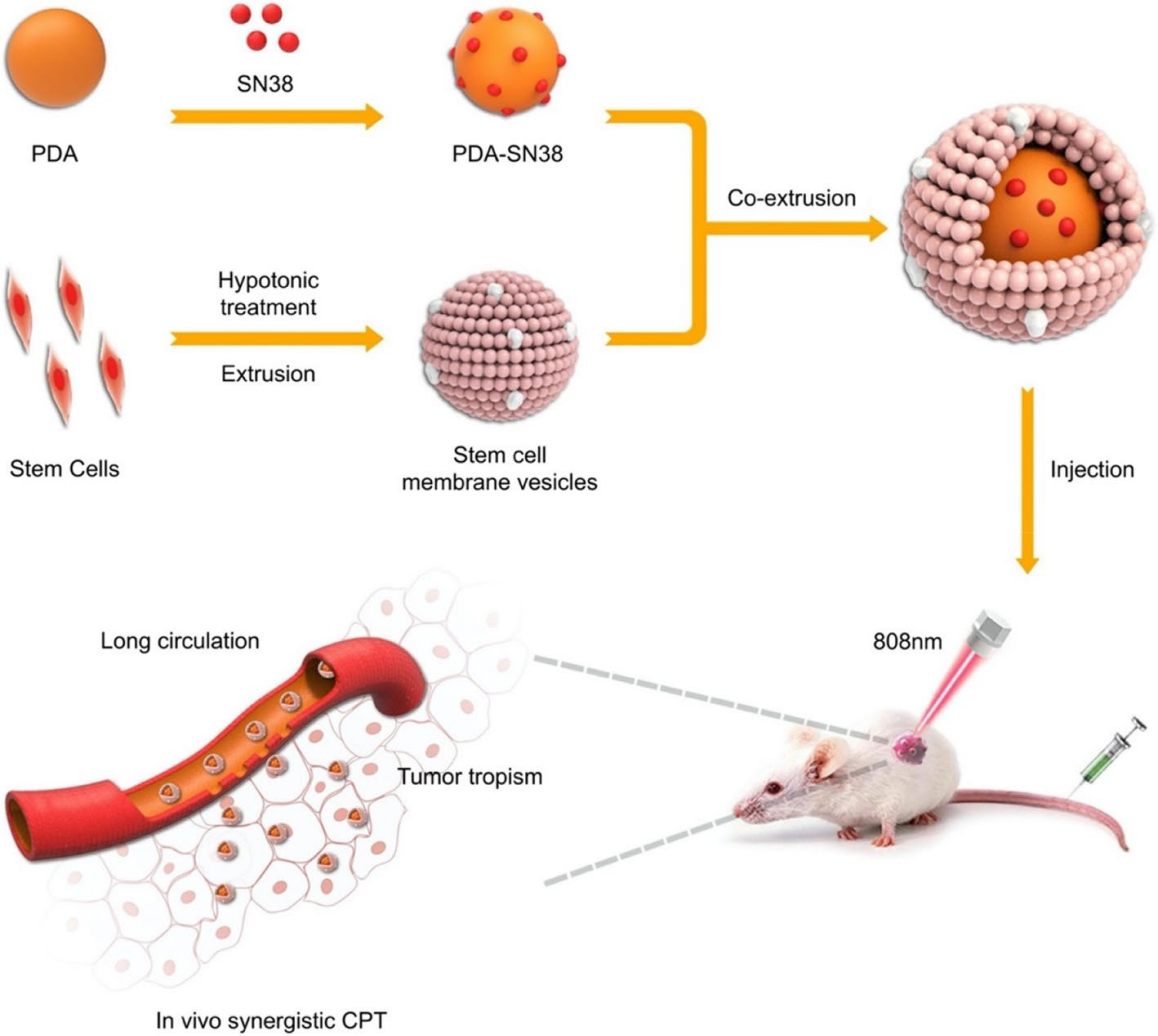
Schematic illustration of the preparation process of PDA-SN38@SCM NPs and its application to CPT of malignant bone tumor. Abbreviation: polydopamine (PDA), 7-ethyl-10-hydroxycamptothecin (SN38).  Reproduced with permission from Ref. [91]

PDA, the polymer of dopamine monomers synthesized through oxidative self-polymerization, contains functional groups such as catechol, amine, and imine [85]. Intensive photothermal conversion efficiency was achievable when photosensitizers were incorporated into PDA with inherent photothermal conversion capability [86]. More importantly, both internal (glutathione) and external (near-infrared light) will trigger the degradation of PDA, achieving controllable drug release [87, 88]. Sun et al. synthesized curcumin (CM)-loaded CS NP by using an ionic gel method, and further functionalized them with PDA coating; the amount of drug release was significantly elevated, which was responsible for the drug delivery behavior by pH/NIR dual-stimulus–responsive of PDA. The high drug release rate at pH 5.5 was more consistent with the procedure in the acidic conditions of TME [89]. In addition to the satisfactorily controlled release of the medicine, accumulating PDA NP in tumor tissues is also essential in PTT, which involves restrictions on the elimination of the reticuloendothelial system and the ineffectiveness of tumor tissue targeting. It can be well addressed by the camouflaged coating of the stem cell membrane (SCM) [90]. Synthesizing PDA-NP camouflaged by the SCM reduced the attachment of biomolecules such as serum proteins to the surface with a durable circulation time in the blood. In contrast to the blood residue rate without SCM-coated NPs (16.7%), a more obvious value was achieved by SCM modification (86.4%), rendering better enrichment in tumors to maximize the tumor-killing effect (Fig. 3). Later, the fluorescence intensity of PDA@SCM NPs in liver, spleen and lung was lower than that of PDA NPs, further confirming that the modification of SCM on the surface of PDA NPs reduced the phagocytosis of NPs by organismal organs [91].

**Fig. 4:**
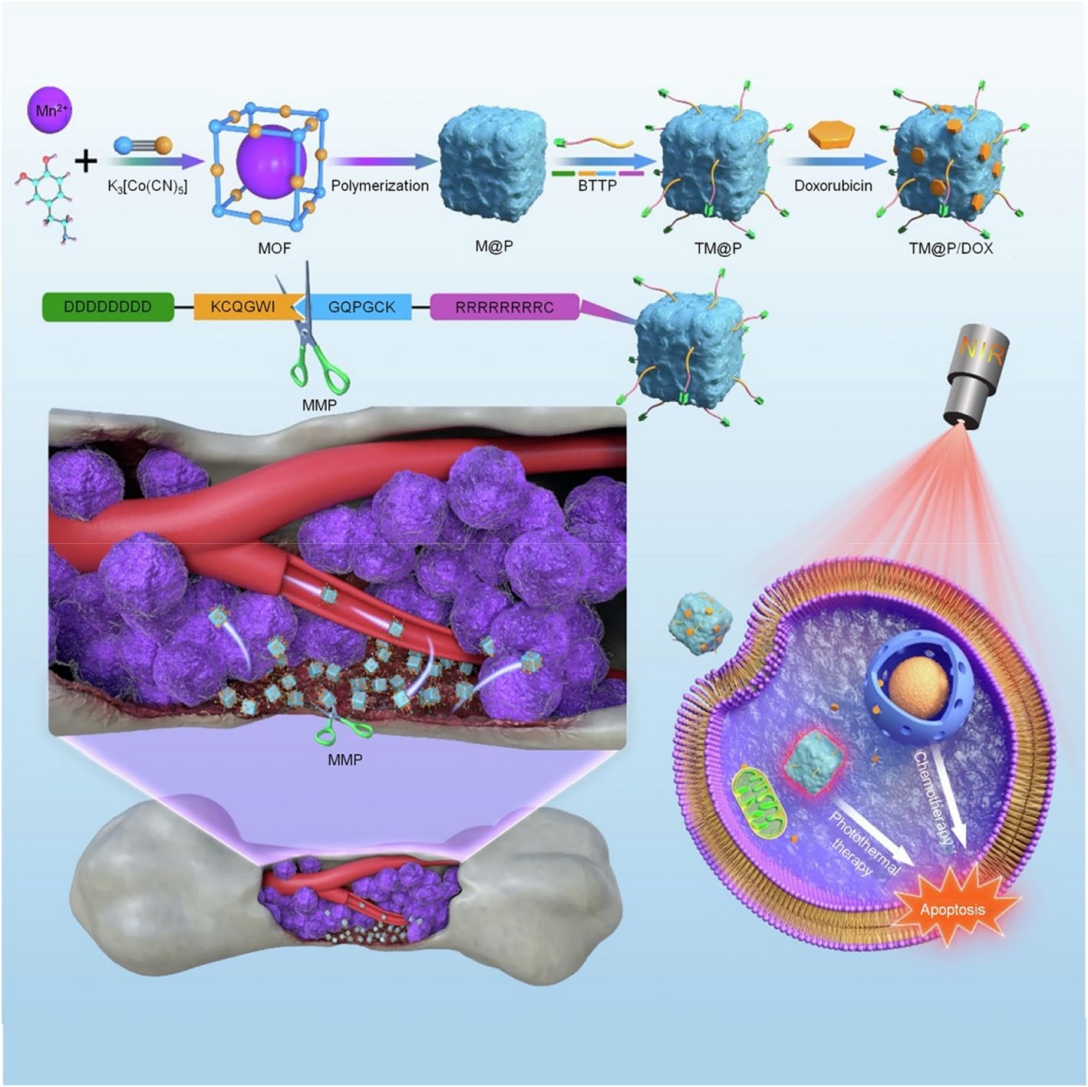
Schematic representation of the BTTP-MOF@PDA/DOX (TM@P/DOX) nanosystem synthesis and the proposed mechanism for the targeted chemo-photothermal therapy (CPT) of bone tumor cells using this nanosystem. Abbreviation: D8-KCQGWIGQPGCK-R8-C (bone tumor cell targeting peptide, BTTP), MOF@PDA (M@P), BTTP-MOF@PDA (TM@P).  Reproduced with permission from Ref. [95]

Not only that, multiple functional groups of PDA permit it to be modified with targeted drugs or peptides, contributing to the precision of PDA nanomaterials for targeting bone tumors [85]. Loading ALN into PDA NP is an appropriate strategy because PDA can carry a variety of drugs and ALN possesses rich groups and targeting capabilities [92]. Therefore, a novel PDA NP was coupled with ALN and modified with DOX, presenting accurate bone targeting properties, photothermal conversion efficiency, drug loading capacity, and multimodal imaging modalities [92]. As described above Asp8 has demonstrated excellent bone-targeting properties, as well as the tripeptide Arg-Gly-Asp (RGD) was extensively used as a tumor-targeting ligand with the capacity for bone remodeling [93]. Kong et al. thereby modified Asp8 and an RGD-derived peptide onto PDA@Gd NP, enabling specific binding to integrin receptors that are overexpressed on cancer cells. Efficient aggregation in the tumor region realized photothermal ablation of tumors, inhibition of osteolysis, and magnetic resonance imaging, developing a versatile theranostic nanoplatform [94]. Recently, a novel bone tumor-targeting peptide (BTTP) was designed to accomplish further improvement in targeting capability. Notably, the KCQGWI-GQPGCK polypeptide fragment of BTTP could be cleaved by matrix metalloproteinases (MMP) secreted by bone tumors after the nanosystem was targeted to the bone interface, followed by exposing a cell-penetrating peptide that directs the nanosystems specifically into bone tumor cells to exert PPT (Fig. 4) [95]. Therefore, it is original for breaking the barrier imposed by the microenvironment on tumor cells by using specific enzymes of the TME, thereby improving the obstacle of difficult targeting of bone tumor cells.

SLN is characterized by adjustable pore size, large specific surface area, and simplified surface functionalization, making them promising candidate materials for PTT in tumors [96, 97]. For example, a super-porous SLN modified by GNPs for delivery of a synthetic short-strand DNA molecule containing an unmethylated CpG motif was used as an adjuvant vaccine for cancer therapy. The nanocarrier with a durable survival rate could deliver CpG and activate the immune system to suppress bone tumor development [98]. Similarly, constructing a homogenous silica nanoplatform equipped with tumor-homing ability, ICG-modified SLN was coated with human osteosarcoma 143B cell membrane, showing excellent stability in the physiological environment and superior specific targeting ability [99]. Notably, in contrast to PDA, non-biodegradability is the most conspicuous flaw in the current research and development of silicon-based nanomaterial therapeutic platforms [100]. However, an organic–inorganic hybrid hollow mesoporous silicone nanocapsule system was synthesized based on the "chemical homology" mechanism, the disulfide bonds (-S–S-) of which could be biodegradable when exposed to the TME. This microenvironmentally responsive disintegration framework provides a platform for rapid drug release, which facilitates the development of novel degradable silica nanoplatforms for PTT of malignant bone tumors [101]. Even so, there was no combination of therapeutic and diagnostic research about nano-silica until Li et al. synthesized a hollow mesoporous silicone nanocapsule that was modified by CuS NP@bovine serum albumin (BSA) through the disulfide bond reaction. The introduction of CuS enabled the silica NP to be operated with photoacoustic imaging, the concealability of which in blood circulation was improved by BSA to avert premature depletion [102].

Hydrophobic drugs are difficult to deliver to the target area efficiently because of their poor solubility [103]. Notably, liposome-mediated NP is responsible to be used as carriers, elevating water dispersion [104]. A liposome-mediated NP platform (IR-7-LIPo/HA-CPG), synthesized by polyvalent immune adjuvant (HA-CPG) and liposome-containing fluorophore (IR-7-LIPo) through the lipid film hydration method, the water dispersity and drug-delivery capabilities of which were remarkably improved, playing roles in eradicating tumors and inhibiting tumor metastasis with the combination of PTT and immunotherapy [105]. To achieve an intensive drug payload without intervention by foreign materials, carrier-free nanomedicine will be available, which is assembled from small-molecule therapeutic drugs. A kind of multifunction NP (MHD-DI) was made up of double-targeted prodrugs (the hyaluronic acid skeleton carries methotrexate and doxorubicin) and small molecule-assembled drugs (DOX-ICG complex). MHD-DI was approximately 200 nm in size and could be lysed by PH/NIR stimulus to accelerate drug release [106]. In conclusion, compared with metal NP, the unique advantages of non-metallic NP in terms of degradability and biocompatibility can provide more options and development trends for the PTT of bone tumors.

### Bioceramics (BC)

BC scaffolds mainly include calcium phosphate scaffolds (including HA, bidirectional calcium phosphate, and tricalcium phosphate [TCP]), calcium silicate (CaSiO_3_) scaffolds, and bioactive glass (silicate, borate, and black glass) [107, 108]. Attractively, BC scaffolds are in accord with the composition of bone tissue and characterized by excellent biocompatibility, osteogenesis ability, and mechanical stability [109]. The photothermal properties of BC scaffolds are predominantly accessed through functionalized modification of photosensitizers albeit some BCs inherently possess weak photothermal properties, followed by NIR- stimuli to promote bone defect repair potentially [110]. Photothermal BC scaffolds permit better rehabilitation of tumor-derived bone defects on account of their physical filling effect compared to administrations of photothermal nanomaterials alone. Two principal strategies are available for the functionalization of photothermal BC scaffolds, including external surface modification and internal homogeneous functionalization [111], and this section will present the advantages of BC scaffolds in the PTT of bone tumor treatment, distinguished by the two modification strategies.

#### External surface modification

Surface modification of BC material is one of the main methods to equip it with photothermal ability [112]. Ma et al. prepared β-TCP scaffolds coated with Cu-containing mesoporous silica (MSN) by spin coating method, the uniform and dense spherical nanolayers formed on the surface of the scaffolds guaranteed photothermal activity under NIR and promoted the expression of osteogenic marker genes [113]. Graphene has a high specific surface area, good cytocompatibility, and excellent thermal conductivity is crucial to enhancing the efficiency of photothermal. The superposition gain of several advantages can be maximized in the BC scaffolds with graphene coating based on excellent thermal conductivity [114, 115]. However, conventional graphene coating methods generally inevitably introduce Mn^2+^ and acids into graphene, potentially challenging biosafety. Notably, chemical vapor deposition is a novel approach that avoids the potential bio-toxicity of residual metal ions and enhances the bonding strength of graphene and β-TCP (G-TCP), which is primarily achieved by the thermal reduction of carbon occurring on the BC surface. Meanwhile, an excellent photothermal effect was shown by G-TCP, causing > 90% death of osteosarcoma cells when exposed to 808 nm NIR irradiation [116]. In addition, bismuth coating BC scaffold can accompany both photothermal anti-tumor effects and osteogenic activity [117]. As mentioned earlier, PDA as photosensitizer is distinguished by biocompatibility, biodegradability, and outstanding photothermal conversion efficiency. Accordingly, BC scaffolds with PDA coating are deemed promising for PTT of malignant bone tumors. 3D printing BC scaffolds with uniform self-assembled PDA nanolayers/calcium phosphate coating can kill bone tumor cells through its controllable photothermal effect and support the adhesion and proliferation of rabbit bone mesenchymal stem cells (rBMSCs), promoting repairing bone defects after PTT [118].

**Fig. 5:**
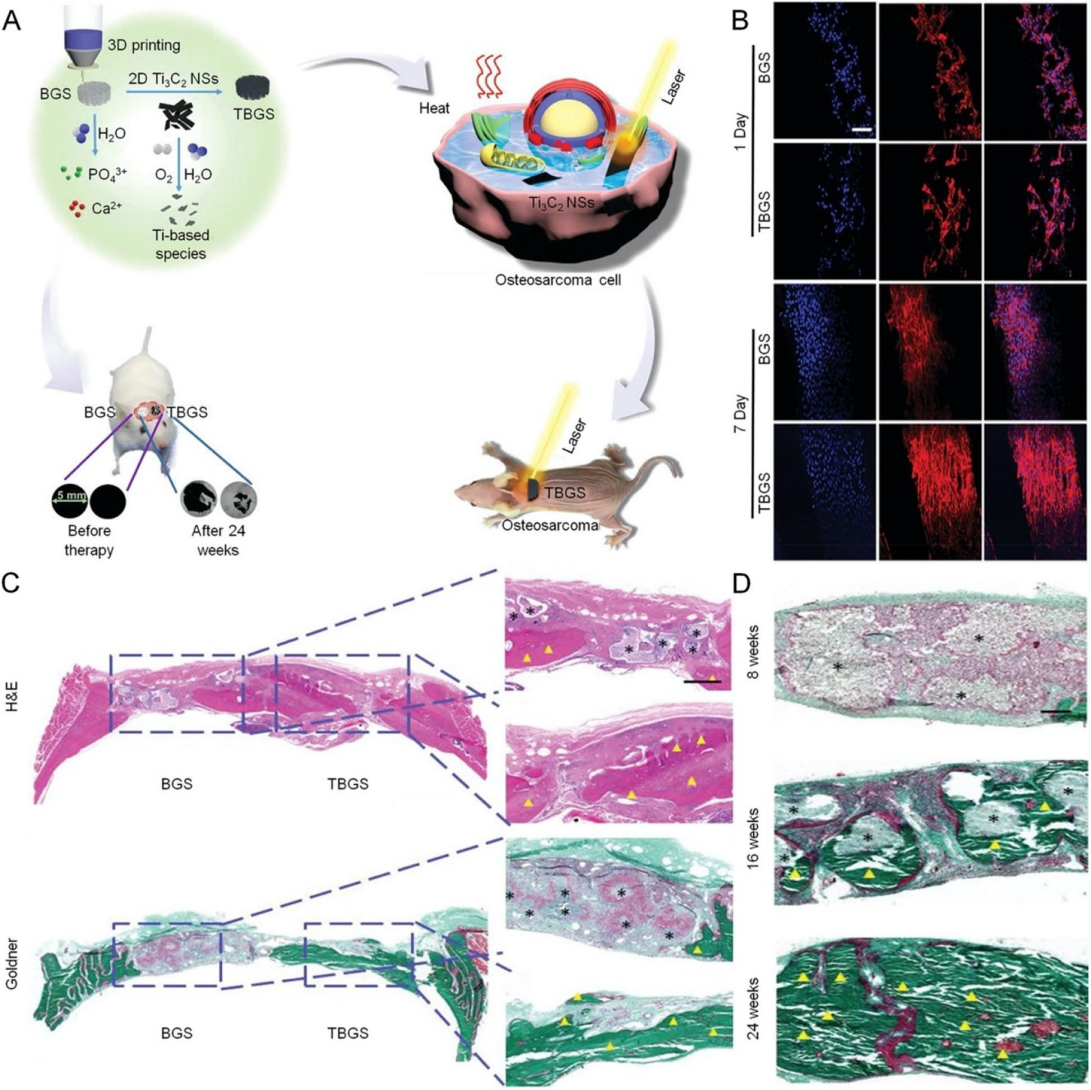
2D Ti_3_C_2_ MXene modified BC scaffold (TBGS) for the PTT of bone tumors and repair of bone defects. **A** Schematic illustration of the synthesis process of TBGS, ablation of bone tumor, and reconstruction of bone tissue. **B** CLSM images of hBMSCs stained with DAPI (cell nuclei, blue fluorescence) and rhodamine phalloidin (cytoskeleton, red fluorescence) on BGSs/TBGSs on days 1 and 7. Scale bar: 100 µm. **C** H&E staining and Goldner staining of rat crania implanted with BGS and TBGS at week 24. Scale bar: 2 mm and 500 µm. **D** Goldner staining of TBGS group at different periods, namely weeks 8, 16, and 24. The defect areas were implanted with BGS and TBGS. Black asterisks mark implanted scaffolds that were not biodegraded completely. Yellow triangles indicate osteogenesis. Abbreviation: Bioactive glass scaffolds (BGS), Ti_3_C_2_-BG scaffold (TBGS), Nanosheets (NSs). Scale bar: 200 µm.  Reproduced with permission from Ref. [123]

Nanosheets (NS) tend to exhibit better photothermal properties than bulk materials when used as coating materials [119]. Recently, MoSe_2_ NS was used to functionalize the surfaces of BC scaffolds for PTT, since Mo and Se are essential trace elements with an active role in human metabolism, facilitating bone regeneration and even photothermal conversion efficiency [120]. Moreover, ultrathin MXene NS is a hybrid of metallic carbide/nitride/carbonitride complex with large specific surface areas and adjustable physicochemical properties [121, 122], which are more applicable as surface modification of BC. Pan et al. have been dedicated to integrating 2D Ti_3_C_2_ MXene with BC scaffolds (TBGS) and implanted the scaffolds into the subcutaneous osteosarcoma model and bone defect model of nude mice respectively [123]. They discovered that bone tumor cells were effectively killed by PTT, and the regeneration rate of bone tissue was accelerated **(**Fig. 5**)**. However, as a type of photosensitizer, Ti_3_C_2_ MXene is mainly confined to the NIR-I with poor tissue penetration depth, while biomaterials belonging to the NIR-II window manifest deeper near-infrared light penetration depth and higher photothermal conversion efficiency, which is convenient to kill deep bone tumors by PTT [124]. Such trouble can be resolved by Nb-based Mxene NS, a kind of BC integrating with 2D Nb_2_C MXene NS that exhibited predominant photothermal conversion capability even in deep tissue within the NIR-II biological window. Moreover, along with NS degradation the Nb-based species being released significantly promoted the neogenesis of blood vessels and bone tissue, and immune cells were simultaneously recruited to the lesion area, accelerating BC degradation to allow new bone growth [125]. Similar NS, 2D borocarnitride (BCN) NS, can interact with Ca ions sites of BC scaffolds and construct hydrogen bondings between BC scaffolds, providing compact coating. Because of the specific photonic response of BCN in the NIR and bone regeneration capacity derived from abundant hydroxyl functional groups and boron elements, an ideal therapeutic effect of PTT on bone tumors and bone tissue mineralization was achieved [126]. Furthermore, because BCN nanosheets are regarded as carbon-based materials that degrade slowly, BCN may degrade and decompose in vivo and ultimately be excreted through the humoral circulation [127]. Copper-coordinated tetrakis (4-carboxyphenyl) porphyrin (Cu-TCPP) NS-modified BC scaffold also expressed effective photothermal ablation and bone regeneration analogously [128].

Adhesion agents are distinctive factors in the modification of BC surface. In a study by Dang et al., poly (_D_, _L_-Lactide) (PDLLA) was therefore adopted as the adhesive material to compactly coat TiN particles on the TCP scaffold [129]. Up to 48% photothermal conversion efficiency of TiN particles endowed the scaffold with excellent photothermal properties, simultaneously the rough surface structure was conducive to cell adhesion as well as with enhanced compressive strength of TCP scaffolds after coating PDLLA. In several other studies by Dang et al., heme particles [130], and LaB_6_ [131], were respectively integrated into 3D printing bioactive glass–ceramic scaffold instead of TiN particles via PDLLA as ever. In conclusion, PDLLA was applied as an applicable medium to tightly combine scaffolds with different therapeutic drugs, guaranteeing efficient utilization of photosensitizers on BC scaffolds and proposing a unique perspective for the development of novel multifunctional scaffolds for PTT. In addition, carbon aerogel (CA) also can be used as a coating with a high specific surface area and enhancing roughness, recruitment osteogenic proteins to repair bone defects. Meantime, this type of carbon-based biomaterial possessed considerable infrared absorbance and laser thermal conversion efficiency, presenting a potentially versatile platform for the healing of post-operative bone defects in osteosarcoma based on the beneficial synergy of the excellent photothermal and osteogenic capabilities of CA coating [132].

#### Internal homogeneous functionalization

Internal homogeneous functionalization can ensure photosensitizer is evenly distributed within BC scaffolds, providing stable efficacy of photothermal conversion, and improving the mechanical properties of BC scaffolds [133]. Yao et al. have dedicated to mixing PDA with HA and carboxymethyl chitosan as the bio-ink to fabricate 3D printing BC scaffolds. The internal PDA particles, HA, and carboxymethyl chitosan were evenly distributed within the scaffold to ensure multiple thermal effects of the composite BC scaffolds and the improvement of mechanical properties [134]. Similarly, a 3D printing porous scaffold was synthesized by graphene NSs incorporated with apatite/gelatin composites, and the proliferation of osteoblasts on it was significantly increased under NIR irradiation, this is not only attributed to similar composition between apatite/gelatin composites and natural bone tissue, but graphene also has good biocompatibility [135]. Surface functionalization is elusive in contrast to the compositional similarity of natural bone tissue exhibited by internal homogeneous functionalized BC scaffolds. In addition, analogous bifunctional scaffolds with osteogenesis were also available by CaTiO_3_ BC scaffolds, providing a promising strategy for the rehabilitation of tumor-induced bone defects and PTT, and the stable photothermal capacity [136].

Akermanite (AKT) BC has been considered to correlate with significantly enhanced compressive strength, can be modified as bifunctional materials for photothermal killing bone tumors and repairing bone defects [137, 138], as well as can be functionalized with photothermal capacity through internal mixing. Moreover, for the reason of porous structures contributing to angiogenesis, cell migration, and nutrient transport, thus porous BC scaffolds would be beneficial to repairing bone defects while killing bone tumor cells through PTT [139, 140]. A free carbon-embedded porous larnite scaffold with uniform interconnected macropores was successfully fabricated, by incorporating silicone in 3D printing CaCO_3_ and converting it to ceramic under an inert gas atmosphere, which exhibited an excellent photothermal efficiency compared with the pure larnite scaffold. Meanwhile, in vitro study presented the expression of osteogenesis genes including ALP, OCN, and Runx-2 were upregulated. The elevated osteogenic differentiation-induced capability was also observed by this modified scaffold in critical-sized rat calvaria defects [141].

### Hydrogel-related biomaterials

Hydrogel is a kind of 3D mesh gel composed of interlaced hydrophilic polymers, which possess water-absorbing ability and interconnected porous structure. Due to their distinct physical and chemical properties, hydrogels generally served as carriers for cells and drug transportation and controlled release [142]. Simultaneously, precise injection into the bone tumor area through minimally invasive approaches could minimize unnecessary damage to normal tissue [143]. Hydrogels can be further presented with photothermal capabilities through the homogeneous incorporation of photosensitizers based on the above advantages. In particular, hydrogels applied as drug delivery systems show significant advantages in PTT [144]. This section summarizes the research on the PTT of bone tumors by modified hydrogels in recent years. Notably, different types of modified hydrogels have obvious discrepant mechanical strength, photothermal conversion efficiencies, and degradable ability, such as cellulose hydrogels [145, 146], agarose-based hydrogels [147], and CS hydrogels [148].

**Fig. 6:**
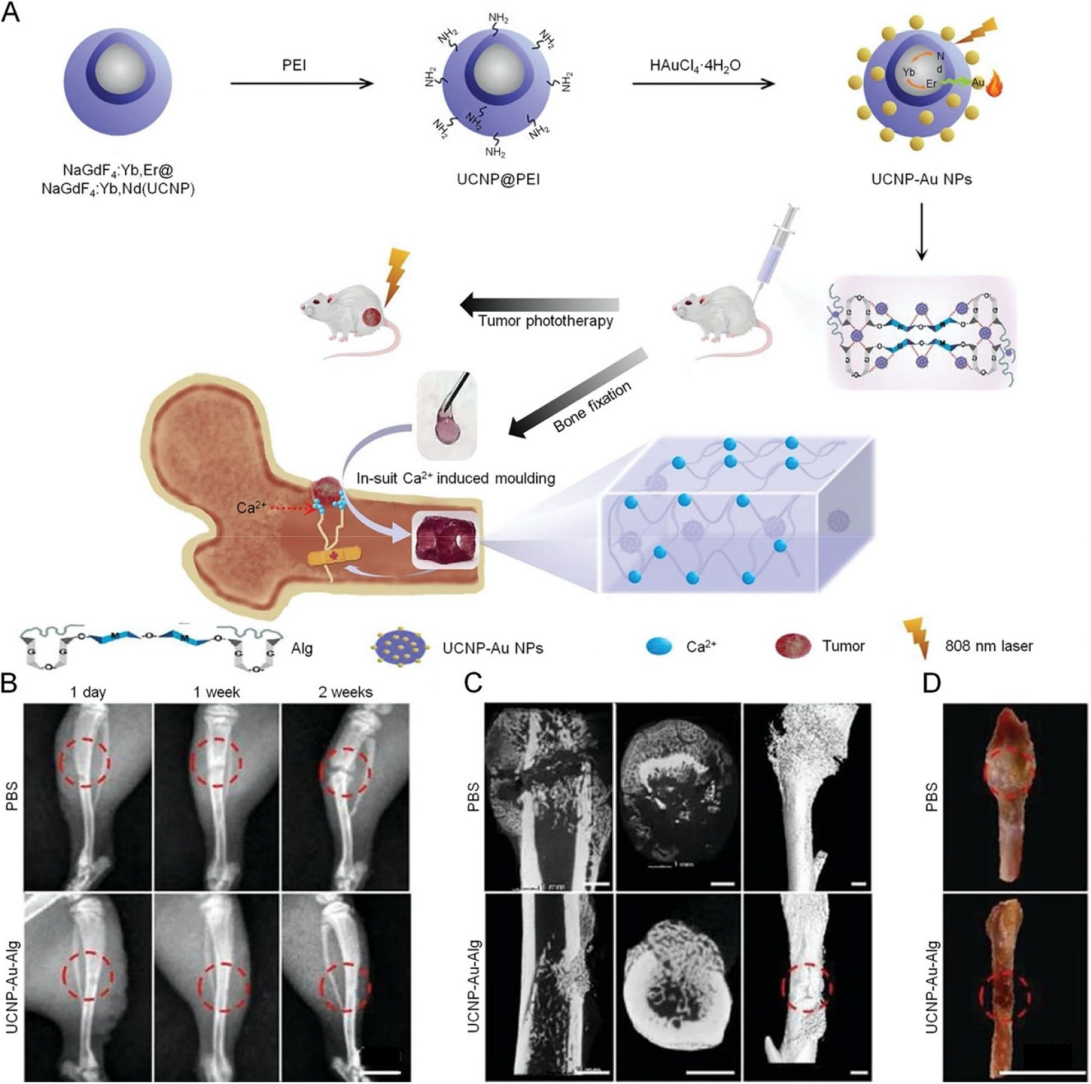
Schematic illustration of the fabrication and application of the UCNP-Au-Alg hydrogel in the PTT of tumor and bone rehabilitation. **A** Injection of UCNP-Au-Alg into the high Ca^2+^ environment of bone tissue accelerated its mechanically stable formation of it. **B** X-ray images from different periods of PBS and UCNP-Au-Alg hydrogel treatment of the tibia, where the red dotted circles represent the distinction between defects. Scale bar: 1 cm. **C** 3D micro-CT images of the tibias treated by PBS or UCNP-Au-Alg hydrogel 14 days after treatment. Scale bar: 1 mm. **D** Pathological sections of tibias treated by PBS and UCNP-Au-Alg hydrogel 14 days after surgery. Abbreviation: NaGdF_4_:Yb, Er@NaGdF4:Yb, Nd (UCNP), polyethyleneimine (PEI), alginate (Alg).  Reproduced with permission from Ref. [151]

Alginate (ALG) is a kind of hydrophilic straight-chain polysaccharide existing in the cell wall of brown algae, which is structurally composed of β-D-mannuronic acid and α-L-glucuronic acid block copolymer, with extensively applied in bone tissue engineering [149, 150]. The simultaneous treatment of tumor removal and bone healing is considered to be a critical proposal for malignant bone tumors. Therefore, through the combination of up-conversion lanthanide-gold hybrid NPs and ALG, a novel near-infrared light-responsive hybrid hydrogel was developed (UCNP-Au-ALG). Under NIR irradiation, the temperature of the hydrogel reached 84℃ with a photothermal conversion efficiency of 36.7%. As shown in Fig. 6, solid tumors were completely eradicated without recurrence by injecting hydrogel into the subcutaneous tissue surrounding the tumor. In addition, the mechanical properties of ALG will be improved to keep the stability of tumor-derived bone defects as much as possible followed by injection into the high Ca^2+^ environment of bone tissue [151]. In terms of biodegradability, an injectable dual crosslinking hydrogel was fabricated based on furan-sodium ALG/bis-maleimide-polyethylene glycol/copper-doped bioactive glass–ceramic microspheres. Owing to the degradable performance of the hydrogel, the photosensitizers were released and degraded into beneficial ions of Si, Ca, and Cu, thereby no bio-toxicity impacted the adjacent tissue, up-regulating the expression of osteogenic genes and significantly promoting the formation of new bone in models of the tumor-derived defect [6].

Gelatin is a hydrophilic colloid of large molecules with poor mechanical properties, the methacrylate group is usually employed as an agent to augment the mechanical properties of gelatin, termed methacrylate gelatin (GelMA) [152, 153]. However, the mechanical properties of GelMA still need to be further improved as biomaterials of bone tissue, additionally, the modification of photothermal properties can be accomplished simultaneously in the process of reinforcing mechanical properties [154]. For example, incorporating black phosphorus (BP) NS into GelMA prepared a therapeutic nano-composite hydrogel with mechanical reinforcement, on account of the intense interactions between the cell-bonding domains of GelMA and the surface charge of BP. Moreover, cancer cells could be eliminated by the prominent photothermal effect of BP within GelMA while inhibiting bacteria with the capability of bone regeneration [155]. The mechanical properties of hydrogel can also be improved by incorporating montmorillonite-strontium into the GelMA while simultaneously purchasing potential bone regeneration [156]. In addition, a bifunctional hybrid hydrogel for the prevention of tumor recurrence and bone rehabilitation was brought into view. Hybridizing Au nanorods and nHA in GelMA /methacrylate chondroitin sulfate hydrogel to fabricate, possessing the significant capability of bone regeneration is attributed to the similarity with the extracellular matrix, which promotes the proliferation and osteogenesis differentiation of MSC [38].

### Other biomaterials

Bone cement, as a well-established commercial polymer biomaterial employed in the orthopedic field, features high strength, injectability, and plasticity [157]. Bone cement can be functionalized with thermogenic ability by incorporating photosensitizers during the polymerization. For example, an inorganic calcium carbonate bone cement with photothermal capability was synthesized by mixing GO particles through co-precipitation. This modified bone cement showed significantly enhanced mechanical properties than pure bone cement for the reason of the charge interaction between tricalcium silicate and GO. As for biocompatibility and osteogenic activity, the proliferation of osteoblasts and the activity of ALP were promoted by this composite bone cement, preserving the photothermal effect of GO simultaneously [158]. Likewise, inorganic calcium phosphate bone cement (CPC) was mixed with cobalt-coordinated tetrakis (4-carboxyphenyl) porphyrin (Co-TCPP) MOF to synthesize composite CPC, possessing excellent photothermal properties. The compressive strength of modified CPC doped with only 1% Co-TCPP was significantly greater than CPC alone. Notably, Co ions released by bone cement could promote angiogenesis in vivo and play a crucial role in promoting bone regeneration. Therefore, this photothermal bone cement with injectability and plasticity has a broad development prospect in repairing critical and irregular bone defects caused by tumor curettage [159].

**Fig. 7:**
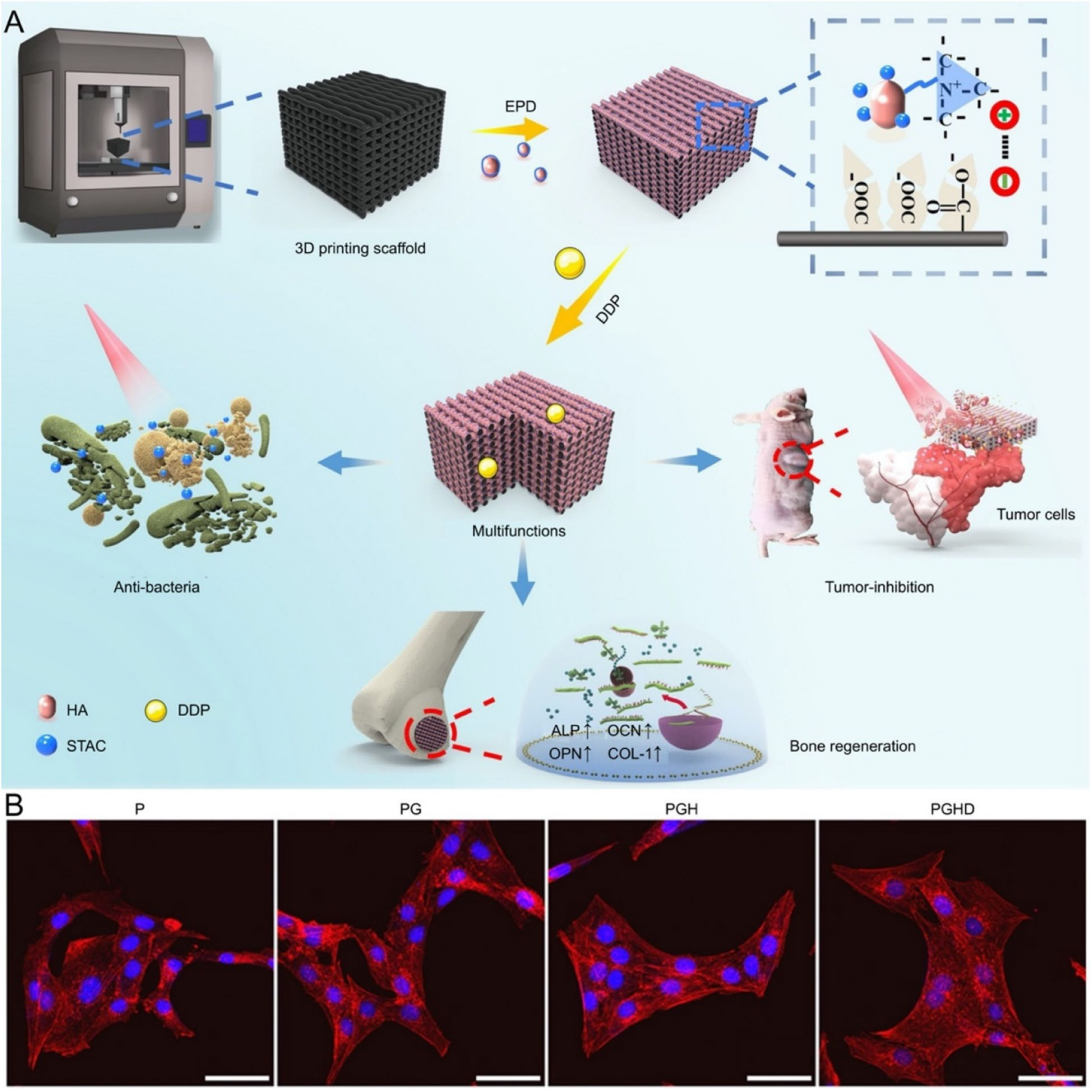
Schematic diagram of the bioactive coating reversing the bioinert deficiency of 3D printing PEEK scaffold. **A** Schematic illustration of the fabrication and associated multi-functions of the scaffold. **B** CLSM images of MC3T3-E1 cells cultured with different scaffolds for 1 day. Scale bar: 50 µm. Abbreviation: electrophoretically deposited (EPD), hydroxyapatite (HA), stearyltrimethylammonium chloride (STAC), cancer drug cisplatin (DDP), PEEK (P), PEEK/graphene (PG), PEEK/graphene/HA (PGH), PEEK/graphene/HA/DDP (PGHD).  Reproduced with permission from Ref. [163]

PEEK, a thermoplastic material with high-temperature stability, has attracted much attention in bone tissue engineering because of its similar mechanical properties to natural bone tissue [160, 161]. PEEK applied for PTT of malignant bone tumors is primarily predicated on photosensitizers modification, providing photothermal conversion ability. Zhang et al. have been dedicated to synthesizing PEEK/graphene nanocomposite, wherein an antibacterial stearyl trimethylammonium chloride-HA layer was coated by electrophoretic deposition on its surface, enabling an active photothermal conversion effect [162]. Moreover, the bioactive coating reversed the bioinert deficiency of PEEK, and effective treatment was put forward by the excellent anti-tumor and anti-bacteriostatic ability. In subsequent research [163], the reliability and development prospect of this 3D printing multi-functional PEEK scaffold was processed revalidation, and the prominent bone regeneration capability of tailored porous scaffolds in combination with the coating was emphasized (Fig. 7). Recently, a layer-by-layer assembled BP-NS/CS composite coating was deposited onto 3D printing PEEK scaffold, achieving on-demand laser-induced heating and drug release. It is of significance that the composite coating augments the biocompatibility of PEEK and the expression of osteogenic-related genes [164]. In addition to PEEK, polycaprolactone (PCL) is another common polymer applied for 3D printing scaffolds as an appropriate substitute for bone repair, similarly, the inherent biological inertia hinders its extensive application [165, 166]. The incorporation of SrCuSi_4_O_10_ NS into PCL to ameliorate its biological inertia is a plausible alternative. In addition, following the degradation of the composite scaffolds, the sustained release of bioactive ions (Sr, Si, and Cu) not only promotes osteogenic differentiation of rBMSCs and angiogenic differentiation of HUVECs in vitro but also enhances new bone formation with increased vascularity in vivo. Moreover, its photothermal conversion efficiency was about 46.3% under NIR-II, and the NIR-II window with more penetrability enabled it to treat deeper malignant bone tumors without side effects than under NIR-I [167].

## PTT-related combination therapy in the treatment of bone tumors

The low-toxicity side effects and simple operating procedure of PTT deserve to be appreciated, while the limited penetration depth of NIR frequently disturbs treatment depth resulting in unsatisfactory treatment effects. In addition, excessive PTT has the potential to generate necrosis and produce a proinflammatory response [168], which subsequently weakens the immune response by inhibiting immune surveillance and immune editing [169, 170]. Notably, the current combination therapy with PTT effectively compensates for the deficiencies of traditional treatment and singular PTT. With the combination of chemotherapy, PDT, CDT, and immunotherapy, impressive improvements in the PTT of malignant bone tumors can be achieved. Moreover, PTT-related combination therapeutic strategies based on advanced materials are different in function while more convenient, safe, and practical than traditional therapy [171]. Therefore, the application of several advanced biomaterials and strategies of combination therapy in PTT for malignant bone tumors are summarized in Table 1. Notably, all the included research in Table 1 was accomplished for both in vivo and in vitro experimentation as studies with both in vivo and in vitro experiments have relatively better scientific credibility.

**Table 1:** Application of several advanced biomaterials and strategies of combination therapy in PTT for malignant bone tumors

Biomaterial	Combination therapeutic strategies	Composition	Type of photosensitizers	Therapeutic agents for bone tumor	Functions	Ref.
NPs	chemotherapy	Fe_3_O_4_ NPs/ZOL	ICG	ZOL-modified PLGA	Magnetic targeting/Bone targeting/Controlled drug release	[22]
	chemotherapy	GNPs/mesoporous silica/MOF/ BYL719/ cisplatin	ICG	BYL719/ cisplatin	Tumor targeting/Photoacoustic imaging	[55]
	PDT	AgBiS_2_ NPs	AgBiS_2_ NP	ROS	CT imaging/Antibacterial	[192]
	immunotherapy	GNR/PEI/cGAMP/anti-PD-1	GNR	cGAMP/anti-PD-1	Reverse immune tolerance	[216]
BC scaffolds	chemotherapy	TCP scaffold/TiN microparticles/DOX	TiN microparticle	DOX	Multiple therapeutic platform	[129]
	PDT	Inorganic CaSiO_3_ bioceramic/Fe	Fe	ROS	Mechanical support/ Osteogenesis	[133]
	immunotherapy	Biodegradable bioglass scaffold/Nb_2_C MXene NS/Mesoporous silica/R837	Nb_2_C@Si NS	R837/anti-PD-1	Robust immune memory	[215]
Hydrogels	chemotherapy	Hybrid methylcellulose hydrogel/Curcumin-loaded microspheres	IR820	Curcumin	Controlled drug release/Osteogenesis	[145]
	chemotherapy	PDA/ n-HA/ cisplatin/oxidized sodium alginate/CS	PDA	Cisplatin	Sustained drug release/Osteogenesis	[148]
	PDT	PNT-gel	ICG	ROS	Controlled release ICG by gel-sol transition	[237]
	antibacterial therapy	GelMA hydrogel/MXene NSs/SP/PDA	PDA and MXene	TOB	Osteogenesis	[238]
Polymers	bone regeneration	CPC/Co-TCPP MOF/GO	GO	Co ions	Osteogenesis	[159]
	antibacterial therapy	PEEK/Graphene NSs/Stearyltrimethylammonium chloride-hydroxyapatite	Graphene NS	Stearyltrimethylammonium chloride-hydroxyapatite	Antibacterial/Osteogenesis/Multiple therapeutic platforms	[162]
	bone regeneration	PCL/SrCuSi_4_O_10_ NS	SrCuSi_4_O_10_ NSs	Sr, Cu, and Si ions	Controlled and sustained ions release/Osteogenesis	[167]

*Abbreviations*: *ICG* Indocyanine green, *ZOL* Zoledronate, *PLGA* Poly (lactic-co-glycolic acid, *BYL719* Alpelisib, *ROS* Reactive oxygen species, *PEI* Polyethyleneimine, *GNR* Golden nanorod, *cGAMP* Cyclic dimeric guanosine monophosphate-adenosine monophosphate, *TCP* Tricalcium phosphate, *R837* An immune adjuvant, *Nb*_*2*_*C* Niobium carbide, *GelMA* Methacrylate gelatin, *SP* Sulfonated polyetheretherketone, *TOB* Tobramycin, *CPC* Calcium phosphate bone cement, *Co-TCPP* Cobalt-coordinated tetrakis (4-carboxyphenyl) porphyrin, *GO* Graphene oxide

### Combination of PTT and chemotherapy

PTT presents a restricted killing effect on tumor cells away from scaffolds, only adjacent tumor cells will be eliminated within an extremely limited distance [172]. Although traditional chemotherapy is a common systemic treatment with obvious side effects, the adverse reactions would be effectively averted if the controllable local release of chemotherapy drugs could be achieved [173]. In addition, PTT is not limited to being a therapeutic method for malignant bone tumors but a trigger for accelerating drug release. Therefore, prospective applications were consequently described by the combination therapy of PTT and chemotherapy, alternatively termed CPT [5, 174].

Constructing biomaterial scaffolds with local drug delivery systems (LDDS) is one of the most frequent combination therapies, persistently releasing chemotherapeutic agents from implanted biomaterials around the tumor area for a durable period. Efficient tumor inhibition and fewer systematic side effects were acquired simultaneously owing to the intensive local chemotherapeutic concentrations [175, 176]. DOX is the most widely used chemotherapeutics in PTT of malignant bone tumors, and NP is often applied as the drug carrier [63, 177]. In terms of excellent killing efficiency, it is emphasized that elevate drug encapsulation and release rate. Therefore, Lu et al. have been dedicated to designing mesoporous silica-coated bismuth sulfide NP with a reasonably distributed mesopore and large specific surface area, wherein the encapsulation of DOX reached 98.5%. The release of DOX would be triggered even under the ultralow-power density of NIR, significantly reducing the systematic side effects of DOX by controllable and responsive release behavior. Such mesoporous NP with a high specific surface area thus is of significance to improve drug encapsulation [178]. In addition to increasing specific surface area, applying adhesion agents is another feasible way to improve drug delivery rate. For example, DOX was attached to the surface of magnesium alloy with PDA as the adhesion agent. The responsive release of DOX under the dual stimulation of heat and pH greatly improved the rate of drug release and therapeutic effect [179]. PDA-coated nanofibrous scaffolds have persistent stable CPT effects as well, with 85.44% drug delivery efficiency and a cumulative drug release of up to 65% for 55 days. NIR and pH dual-responsive drug release properties enabled excellent CPT performance for malignant bone tumors [180]. In several articles by Dang et al., PDLLA was introduced as the appropriate adhesion agent to combine scaffolds with different types of therapeutic drugs, providing a unique insight into the development of CPT [129, 130].

**Fig. 8:**
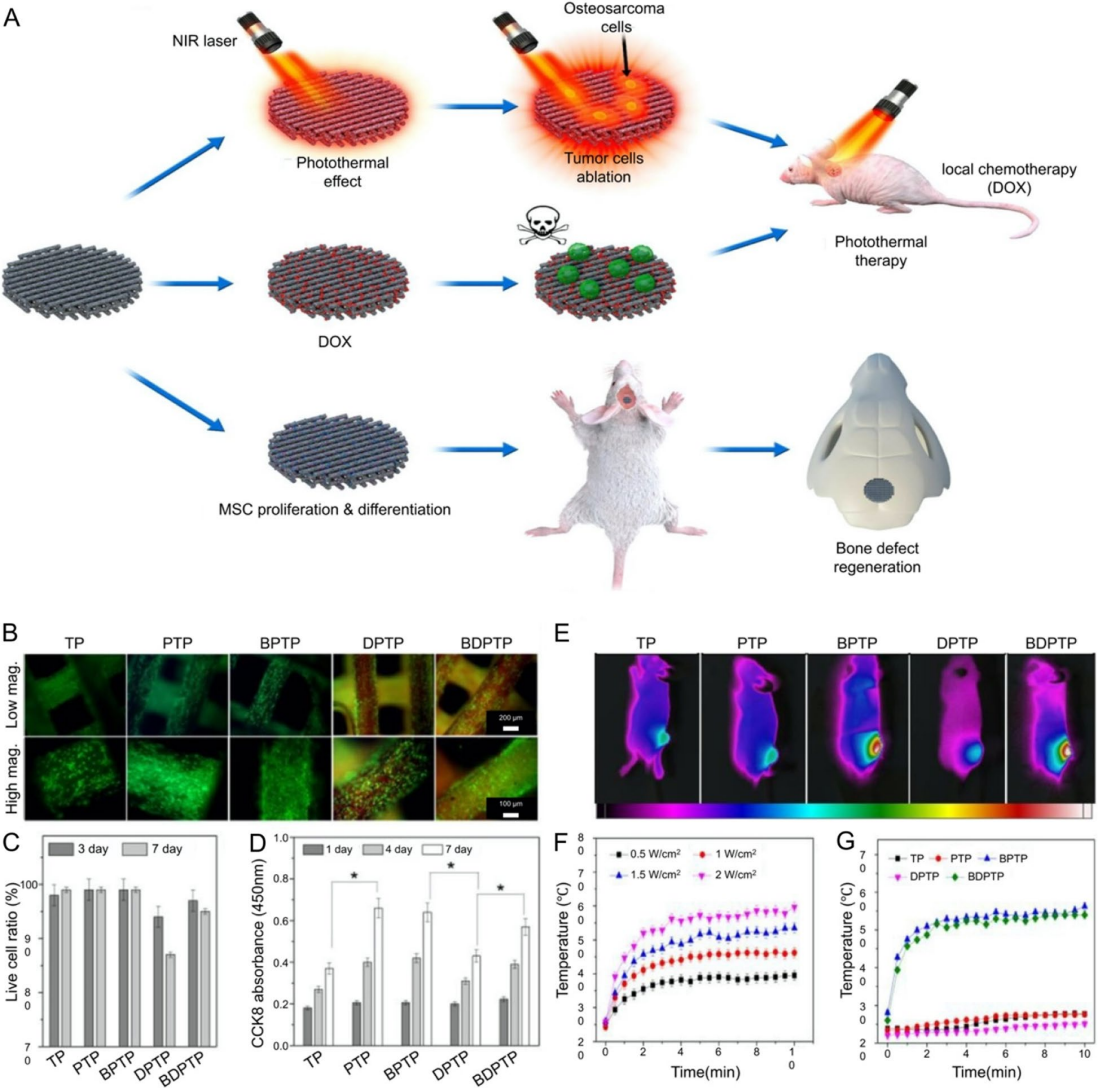
Schematic diagram of “first kill and then regenerate” by CPT. **A** Schematic illustration of a cranial defect in a rat with a multifunctional scaffold implanted by PTT ablation of a tumor in a nude rat, local chemotherapy, and reconstruction. **B** Live and dead images of rBMSCs were cultured on different scaffolds on day 3. Scale bars: 200 µm and 100 µm. **C** Viability of rBMSCs on days 3 and 7. **D** The proliferation of rBMSCs on scaffolds in the 7 days. **E** Temperature distribution of tumor tissue implanted with scaffolds in vivo. **F** Effect of NIR laser power on the temperature increase of BDPTP scaffolds. **G** Effect of temperature increase of different scaffolds irradiated by NIR laser. For panels **C** and **D**, *p < 0.05. Abbreviations: mesenchymal stem cell (MSC), black phosphorus (BP), tricalcium phosphate (TCP), poly (lactic-co-glycolic acid (PLGA), TCP/PLGA (TP), P24/TCP/PLGA (PTP), DOX/P24/TCP/PLGA (DPTP), BP/P24/TCP/PLGA (BPTP), DOX/P24/BP/TCP/PLGA (BDPTP).  Reproduced with permission from Ref. [187]

The negative impact on osteogenesis from exorbitant concentrations of chemotherapeutic drugs desiderates to be more attention [181, 182], on account of reconstructing skeletal stability is essential after the curettage of bone tumor [183, 184]. Nevertheless, the significance of reconstructing the skeleton has been objectively balanced in some studies of CPT for malignant bone tumors [141]. A multifunctional porous scaffold composed of M-type ferrite particles (SrFe_12_O_19_), mesoporous CaSiO_3_, and CS, which prominently promote osteogenesis and the effect of CPT against osteosarcoma, the osteogenic effect of which is primarily attributed to the magnetic properties of SrFe_12_O_19_ [185]. Similarly, excellent therapeutic effects were presented by a type of PDA NP loading ALN, by carrying osteogenic drugs to reverse the unbalanced microenvironment of bone destruction [92], and effectively inhibits osteolysis [186]. As previously described, ALN elevated the accuracy of targeting and possessed an obvious osteogenic-promoting effect simultaneously, without significant toxicity to any major metabolic organ via evaluating hematological toxicity and liver function. Therefore, a clinical treatment modality of residual osteosarcoma after repairing bone defects was provided by ALN-mediated CPT [92]. As well as reducing the toxicity of drugs is crucial in promoting osteogenesis, and BP NS could significantly reduce the long-term toxicity of continuously released DOX during bone regeneration in vivo [187]. Therefore, a hierarchical porous nanocomposite scaffold was cryogenically 3D printed by incorporating β-TCP, 2D BP, DOX, and high-dose osteogenic peptides. Based on the synergistic effect of chemotherapy and PTT, the tumor was inhibited at the first stage, then the reconstruction of bone defects was achieved by the continuous release of osteogenic peptides in succession (Fig. 8) [187]. Notably, conceiving the clinical perspective on the rehabilitation of bone defects after surgical resection of bone tumor, the perspective of “first kill and then regenerate,” a step-by-step approach to tumor killing followed by bone repair is extremely meaningful.

### Combination of PTT and photodynamic therapy (PDT)

PDT involves regulating the microenvironment of tumor cells by producing abundant reactive oxygen species (ROS) and hydrogen peroxide (H_2_O_2_), characterized by local photodamage to the solid tumor area itself [34, 188]. Notably, both PTT and PDT are stimulated by NIR irradiation, generating double benefits of tumor-killing by the single irradiation wherein the synergistic effect of ROS and hyperthermia are taking effect.

The mild thermal effect generated from PTT can augment the photosensitizer absorption of cells, simultaneously improving the efficiency of PDT by the intensive concentration of photosensitizers [189]. In terms of endocytosis, a kind of mesoporous bioactive glass doped with manganese (Mn) and loaded Ce6 into the mesoporous channel (5Mn-MBG/Ce6) possessed promoted uptake efficiency by cells when exposed to NIR, accumulating extensive concentrations of Ce6 in cells to generate more ROS. The efficiency of PDT has been greatly improved with the assistance of adequate ROS [190]. Similarly, nano bismuth (Bi) is also characterized by high ROS production when exposed to NIR [191]. A kind of AgBiS_2_ NP was fabricated by a facile solvothermal method, which exhibited CT contrast at the tumor site and excellent effects of PDT simultaneously with sufficient ROS after a relatively extended period of NIR exposure [192]. However, the hemangiectasis and local blood transport of tumors will be increased after long-term heating, which may amplify the possibility of tumor metastasis [193]. Accordingly, the high yield of ROS is the prerequisite for reducing the duration of hyperthermia even the risk of metastasis. A NIR triphenylamine-grafted boron dipyrromethene derivative was capable of producing plentiful ROS, with even 35.2% of singlet oxygen generation efficiency [194]. The further study, NPs doped with fluorine, PDA, and collagen in titanium dioxide (TiO_2_) could kill osteosarcoma cells within 10 min by the ROS produced under NIR irradiation. Moreover, the proliferation and differentiation of BMSCs were facilitated by the synergistic impact of PTT and PDT [195]. For efficient ROS production, a better alternative was offered by optically active semiconducting polymers [196], which exerted multifunction in aiding the diagnosis, treatment, and prognosis of osteosarcoma [197]. Discrepant therapeutic diagnostic effects could be presented under different NIR biological window (NIR-I/NIR-II) irradiation, providing fluorescent emission in the NIR-II and photoacoustic signal in the NIR-I. In addition, the semiconducting polymer NP with good photodynamic conversion efficiency could also enable CPT through carrying chemotherapeutics [197].

**Fig. 9:**
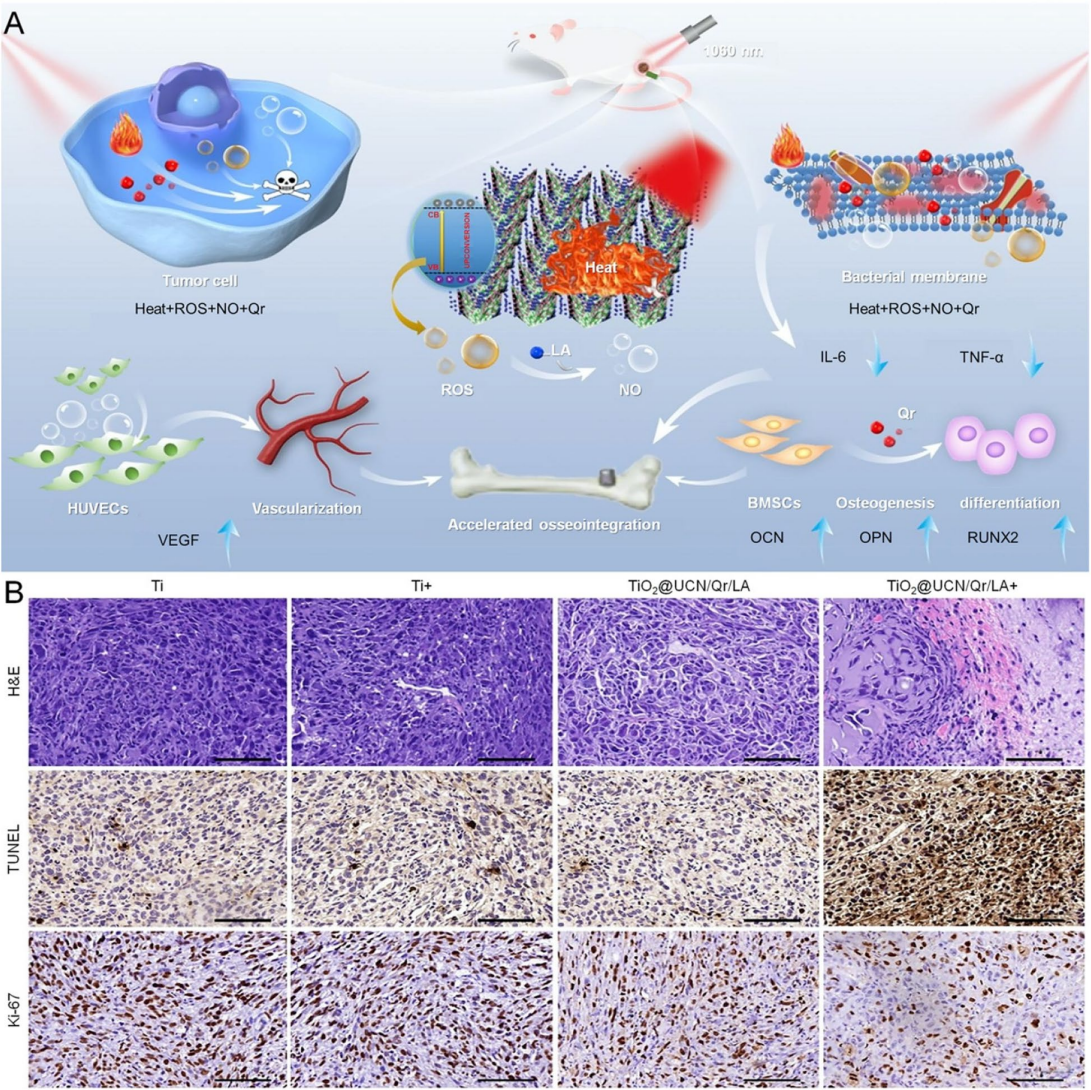
Mechanism diagram of NO treatment combined with PTT. **A** Schematic illustration of TiO_2_@UCN/Qr/LA nano-shovel on Ti implants for tumor ablation, biofilm elimination, vascularization, and bone regeneration. **B** H&E, TUNEL, and Ki-67 staining of tumor sites on day 15. Scale bar: 200 µm. Abbreviation: Quercetin (Qr), L-Arginine (LA), human umbilical vein endothelium cells (HUVECs), vascular endothelial growth factor (VEGF), bone marrow mesenchymal stem cells (BMSCs), titanium dioxide nano-shovel/quercetin/L-arginine (TiO_2_@UCN/Qr/LA), Osteogenesis-related gene (OCN, OPN, and RUNX2).  Reproduced with permission from Ref. [203]

Nitric oxide (NO) exerts diversified functions in many physiological and pathological circumstances, constantly associated with ROS [198, 199]. The high level of NO is inclined to pro-oxidative cytotoxic effects, which in reaction with ROS generates cytotoxic peroxynitrite (ONOO^−^) and other reactive nitrogen species (RNS), thereby impairing the function of biological macromolecules [200]. Lee et al. designed a diethylenetriamine/nitric oxide adduct-loaded polylactic acid combined with ICG and used it as an exogenous NO donor. ROS produced by ICG reacted with NO and release ONOO^−^, causing multiple biomolecular damages to osteosarcoma cells [201]. Similarly, a 2D Nb_2_C MXene-wrapped 3D printing BC scaffolds coating mesoporous silica possessed prominent photothermal conversion efficiency and productivity of NO, thereby elevating the anti-tumor effect. It is also has been demonstrated that high concentrations of NO released in the first phase enable anti-tumor properties, and low concentrations of NO in the later phase enhance vascular regeneration and bone regeneration in the scaffold [202]. The uniqueness of the NO treatment mechanism combined with PTT deserves to reference. As shown in Fig. 9, a composition of rare-earth elements-saturable titanium dioxide nano-shovel/quercetin/L-arginine (TiO_2_@UCN/Qr/LA) was modified on titanium implant, facilitating the generation of ROS when exposed to NIR-II. ROS reaction with LA can subsequently release NO, thereby exhibiting desirable inhibition of osteosarcoma. Moreover, additional functions including infection prevention and angiogenesis promotion were also presented [203].

Because LA catalysis with ROS can produce numerous NO free radicals to assist in tumor killing [204], it is a feasible method to obtain more NO free radicals consequently by adding LA onto orthopedic implants. An LA-modified mesoporous nanocomposite was developed as a therapy platform, applying for the generation of NO triggered by ROS to coordinate the efficacy of PTT. Its excellent photothermal conversion efficiency, ROS production rate, and concomitant NO release ability make it an ideal biomaterial for killing osteosarcoma cells [205]. Mitochondrial apoptosis is an inevitable sign of tumor necrosis, with activating of the caspase cascade [206, 207]. Therefore, Zeng et al. prepared a modified GO nanocomposite to target mitochondrial, eradicating tumors. The synergistic therapy of PTT and PDT was realized under NIR irradiation simultaneously inhibiting adenosine triphosphate synthesis after NP accumulation in the mitochondria. Drug resistance and growth of bone tumors can be reduced by inhibiting the mitochondrial function of tumor cells [208]. In conclusion, PDT and CPT are effective therapeutic methods based on PTT, actively mobilizing components of the TME to kill tumors and reduce the possible side effects of the additive as well as providing a prospective treatment program.

### Combination of PTT and immunotherapy

The tumor-specific neoantigen mutation will contribute to the resistance of chemotherapeutics [209, 210]. However, tumor immunotherapy does not directly attack tumor cells, but rather induces an immune response from the body’s immune system [211]. The tumor immunological response includes cellular humoral immunity, among which cellular immunity plays the most significant role. T cells, DCs, macrophages, and NK cells are all involved in anti-tumor cellular immunity [212, 213]. As a therapeutic approach, PTT can induce the apoptosis of tumor cells rather than necrosis. Moreover, the antigen fragments generated by tumor cell apoptosis will be presented to the DCs to achieve recruitment and maturation, activating plenty of immune T cells consequently in the tumor environment [214]. Therefore, based on the immune regulation ability of PTT, in combination with immunotherapy will observably enhance tumor suppression. Thereby, this immunosuppressive microenvironment could be profoundly reversed under the influence of the photothermal effect [75, 98].

**Fig. 10:**
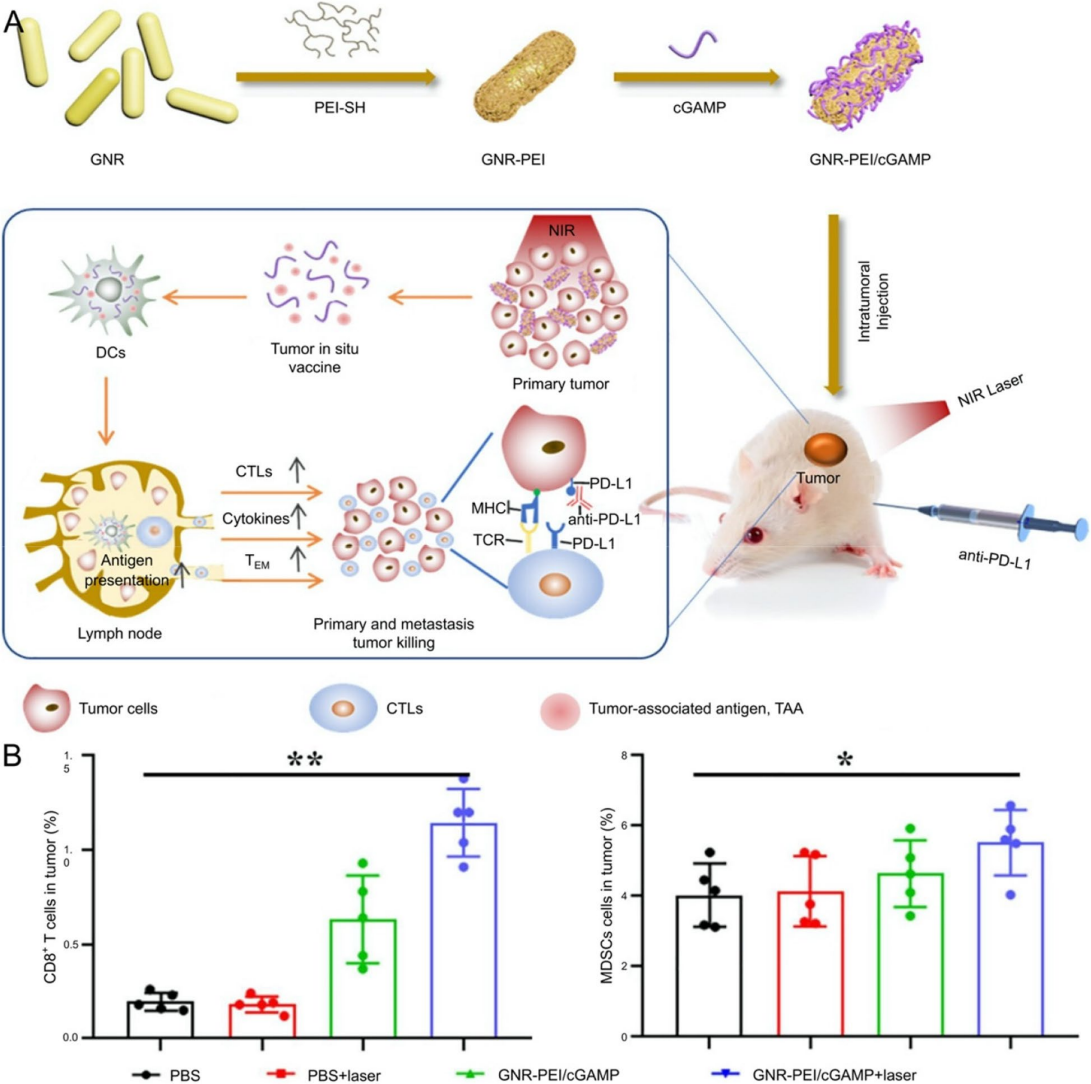
Schematic representation of the biomaterial design mechanism for the combination of PTT and immunotherapy. **A** Schematic illustration for the synthesis of GNR-PEI/cGAMP, the combination of localized tumor vaccine and anti-PD-1, the checkpoint blockade and tumor immune tolerance microenvironment reversed. **B** The proportion of CD8.^+^ T cells and MDSCs in distant tumors. Abbreviation: golden nanorod (GNR), thiolated PEI (PEI-SH), PEI conjugated GNR (GNR-PEI), cyclic dimeric guanosine monophosphate-adenosine monophosphate (cGAMP), dendritic cells (DCs), cytotoxic T lymphocytes (CTLs).  Reproduced with permission from Ref. [216]

The immune capacity of the organism can be appropriately mobilized by PTT combined with immunotherapy, avoiding dependence on additional therapies [211]. For instance, a niobium carbide (Nb_2_C) modified novel 3D printing scaffold (BG@NbSiR) was implanted, followed by injecting anti-programmed cell death protein-1 ligand (PD-L1) to regulate the TME. Notably, the primary and metastatic tumors were eliminated concurrently, furthermore, establishing a persistent immune memory in the host body to prevent tumor recurrence [215]. Although the methods of establishing bone metastasis and recurrent models did not comprehensively comply with the practical clinical situation, this research was significant in treating bone metastasis of breast cancer, involving biosafety, tumor elimination, long-term immune memory, and significant osteogenic properties of the above. In contrast, a composite of S–Au conjugated polyethyleneimine (PEI) and golden nanorod (GNR), interacting with cyclic dimeric guanosine monophosphate-adenosine monophosphate (cGAMP) through electrostatic (GNR-PEI/cGAMP), generating thermal in situ immune vaccines to recruit DC-capturing tumor-associated antigens [216]. Amplification and activation of dendritic cells (DC) could be further stimulated by cGAMP, motivating the specific immune killing of cytotoxic T cells. The predominant mechanism is associated with the blockage of immune checkpoints and reversion of tumor immune tolerance microenvironment by immune regulation (Fig. 10). Inspired by tumor immunity induced by DC, Sun et al. developed intelligent organic DCs (iDCs), which could stimulate T cells in the tumor to secrete cytokines in situ. T cells would be activated when iDCs entered cells of lymph nodes, then migrate to the tumor site and secrete cytokines to reduce the HSP expression of cancer cells, in which the sensitivity to heat stress of cells was increased, reversing the immune suppression of the TME [217]. Therefore, enhancing the maturation of DCs is essential for improving the immunotherapeutic capacity, and improving the infiltration of cytotoxic T lymphocytes in primary and distant tumors [218]. Under NIR, a new nanoplatform could trigger the activation of innate immunity through the cGAS-interferon gene signaling pathway, releasing immune adjuvant, and tumor cell protein antigens to reinforce adaptive immunity simultaneously [219]. Notably, the synergistic effect of innate and adaptive immunity in the presence of PTT promoted the maturation of DCs, activating a virtuous cycle of cancer immune through the dead tumor cells and viable immune cells, contributing to the controlled release of immune adjuvant and the infiltration of cytotoxic lymphocytes into the tumors.

Tumor-associated macrophages (TAM) also play a vital role in tumor inhibition. M1 and M2 are two different polarization states of TAM. M1 act in an anti-tumor role, while M2 is inclined to promote tumor growth and invasion [220, 221]. In recent research, GO and polyethylene glycol was used as photosensitizers, inducing macrophages to differentiate into the M1 phenotype and inhibiting the differentiation process of the M2 phenotype by PTT. Therefore, once TAM was induced into an optimal M1 polarization state, providing a prominent anti-tumor effect. The theoretical basis demonstrated by this study provides a new idea for PTT combined with immunotherapy [222].

### Combination of PTT with other treatments

Chemodynamic therapy (CDT) is a therapeutic strategy for regulating TME, primarily relying on the Fenton response in the presence of ferrous or transition metal ions [223, 224]. Both NIR and heat will accelerate the Fenton reaction, improving the effect of CDT, which are definitely advantages that deserve to be sufficiently applied in PTT [225, 226]. To achieve prominent synergistic therapeutic efficiency of PTT, PDT, and CDT, Prussian blue analog (PBA) NPs with extensive absorption efficiency were introduced. CDT inhibited tumor invasion through the regulation of the epithelial-mesenchymal transition process, providing a novel direction for the photothermal combination of CDT in the treatment of malignant bone tumors [227]. For cascading the effects of PTT, CPT, and CDT therapeutic modalities, a nanoplatform was designed and could be modulated by the TME. Notably, the boronate–catechol linkage of it could be cleaved when in acidic and H_2_O_2_ over expression TME, thereby chemotherapeutics and Fe^2+^ were released to initiate CPT and CDT [228]. Considering the TME could be modulated by CDT, a nanomedicine based on oxygen-perfluorotributylamine was composed of a PDA-coated UIO-66 MOF as the drug carrier to load TPZ, activating the oxygen-dependent HIF-1α pathway when exposed to NIR, further enhancing hypoxia in the TME to induce apoptosis of osteosarcoma cells subsequently [229]. CDT is an effective treatment, however, its therapeutic impact is severely restricted by the overexpression of GSH [230]. To resolve this dilemma, an excellent nanocatalytic platform consumed GSH through the release of Fe^3+^ and Cu^2+^ mediated redox reactions in the presence of NIR, substantially intensifying CDT efficacy and the anti-osteosarcoma effects of synergistic CDT/PTT/CPT [231].

**Fig. 11:**
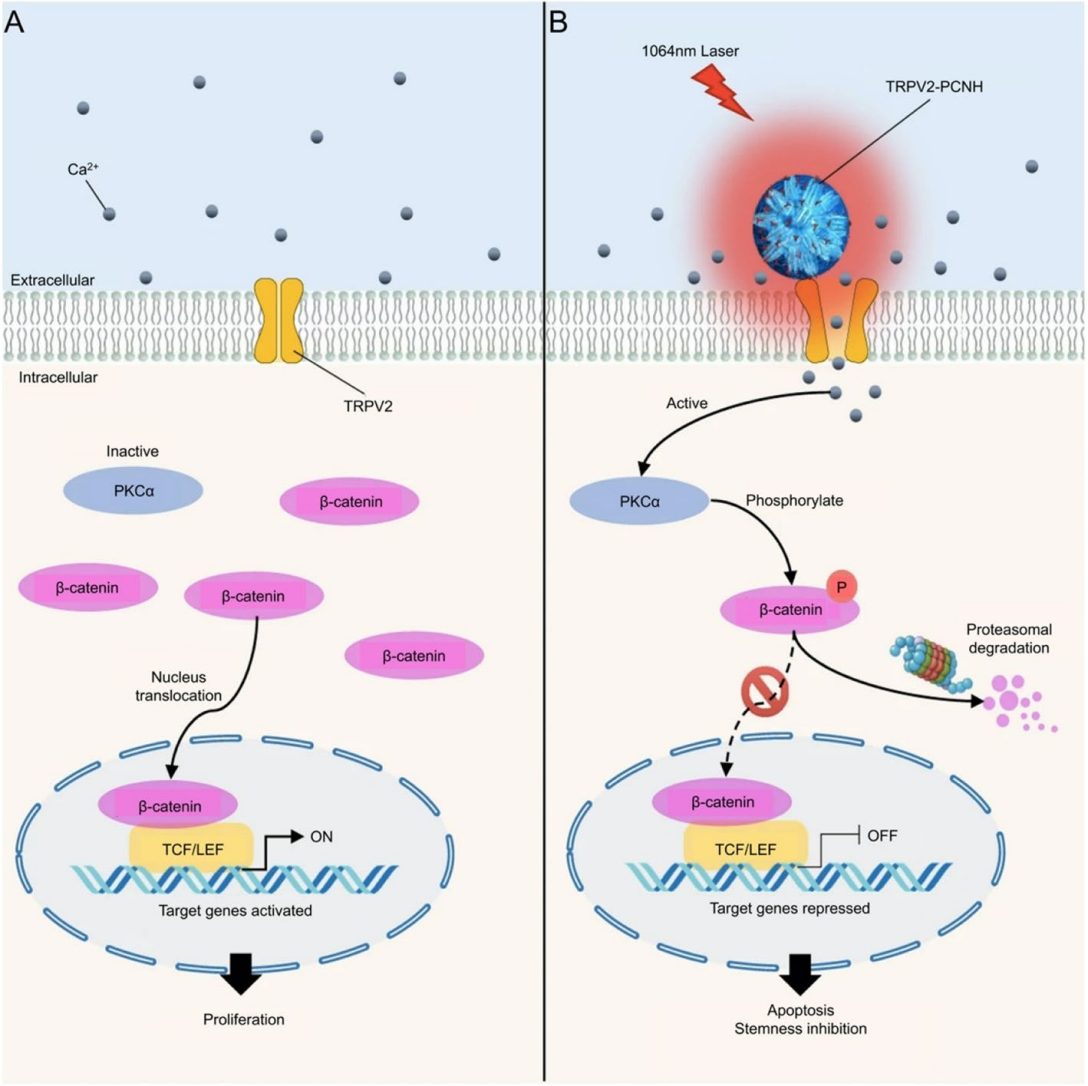
Schematic illustration of the mechanism for eradication CSCs by near-infrared photoactive carbon nanohorn (CNH) complexes; (**A**) In the absence of stimuli, transient receptor potential vanilloid family type 2 (TRPV2) channels are maintained in the off-state to maintain intracellular Ca^2+^ homoeostasis. PKCα is inactivated because of low concentrations of cytosolic Ca^2+^. β-catenin that is stabilized and accumulated in the cytosol translocates into the nucleus and activates its target genes, leading to cancer proliferation. **B** In the presence of stimuli, antibody-guided CNH targets TRPV2 receptors and activates TPRV2 channels through the heat generated from laser radiation. Ca^2+^ influx via TRPV2 channels increases PKCα activity, leading to β-catenin phosphorylation. This phosphorylation promotes rapid degradation of cytosolic β-catenin by the proteasome. Thus, the expression levels of genes that are involved in cell survival and stemness are repressed, resulting in apoptosis and inhibition of cancer stemness. Abbreviation: a protein kinase that directly phosphorylates β-catenin in the presence of Ca^2+^ and promotes its degradation (PKCα), PEGylated CNH (PCNH).  Reproduced with permission from Ref. [233]

Notably, cancer stem cells (CSCs) are mainly responsible for contributing to drug resistance and leading to recurrence, or metastasis [232]. To eradicate CSCs, a NIR photoactivated carbon nano angle (CNH) complexes could be driven to disrupt intracellular Ca^2+^ homeostasis of CSC when Ca^2+^-dependent CNHs were degraded under NIR. Carcinogenic Wnt/β-catenin signaling was consequently triggered to inhibit tumor cells as shown in Fig. 11 [233]. From this, oxygen-independent free radical-based thermotherapy is of great significance in treating bone tumors under hypoxic conditions. Fortunately, thermodynamic therapy (TDT) presented prospective applications in hypoxic tumor treatment based on oxygen-irrelevant free radicals [234]. But overexpression of glutathione (GSH) in tumor cells scavenges free radicals, significantly reducing the therapeutic effect of TDT [235]. Hu et al. conceive of accumulating free radicals in mitochondria to consume redundant GSH, enhancing the synergistic effect of PTT and TDT [236]. A hollow mesoporous MnO_2_ nanoplatform was accordingly designed and applied by them, oxidizing intracellular GSH to glutathione disulfide (GSSG) by MnO_2_ under NIR irradiation; then, abundant free radicals were accumulated in the mitochondria to reduce the mitochondrial potential, contributing to mitochondria-mediated apoptosis of tumor cells.

## Conclusion and future perspectives

Given the complex pathogenesis and insidious disease progression of malignant bone tumor, the long-term survival rate and end-stage life quality of patients are unsatisfactory. Moreover, chemotherapy, curettage, and other traditional primary treatments accompany problems such as damage to normal bone tissue, tumor recurrence, and systemic toxic side effects. Nevertheless, the iterative innovation of PTT-related biomaterials and the continuous improvement in combination therapeutic strategies has brought a better opportunity for the radical elimination of malignant bone tumors in the future, which is exciting news for patients.

The modifying methods and features of advanced photothermal biomaterials have been adequately introduced in this review. Nanomaterials are accepted to be the most familiar photothermal biomaterials based on inherent advantage properties. In addition, BC scaffolds are capable of photothermal through surface coating or internal homogenization of photosensitizers, simultaneously osteogenesis will be promoted by adjusting the mesoporous materials, roughness, and surface area. Although hydrogels are not applicable as a substitute for bone tissue without desirable mechanical properties, excellent thermal therapeutic effects can be simultaneously present with modified high-intensity hydrogels. Furthermore, some other biomaterials can retain biological activity, mechanical properties, and chemical properties while producing efficient photothermal effects. The strategy of combination therapy is an appropriate treatment applied for reducing drug toxicity, elevating tumor-killing efficiency, stimulating anti-cancer immunity, and amplifying immune sensitivity. Improving the drug delivery rate and release rate, ROS production efficiency, and activation of immune cells will constantly enlighten further research in promoting PTT-related combination therapy. Furthermore, a solid foundation for improving the efficiency of PTT-related combination therapy in bone tumors has been established and has the potential to transform into clinical treatment in the near future.

Further improvements are of necessity in photothermal biomaterials and combination therapeutic strategies in the aspects of wavelengths of NIR. Notably, restricted by the NIR laser penetration depth and the distance from the body's surface to the bone, the effectiveness of photothermal therapy in animal experiments differs from its clinical application. Therefore, accelerating the technological maturity of photothermal therapy and facilitating its clinical translation is an imperative ongoing direction. Involving various photosensitizers requires discrepant wavelengths of NIR for heat production and most photosensitizers have weak photoresponsivity at deeper sites, thereby elevating the photothermal conversion efficiency of photosensitizers to exert hyperthermia in deep bone tumor tissues is crucial. Moreover, the potential or underestimated negative effects of photothermal biomaterials on bone tumor therapy deserve attention and further evaluation. Most scientists are consistently emphasizing the advantages and attractions of biomaterials in PTT of bone tumors, despite without significant side effects, but the fate of biomaterials after PTT application is often neglected, including biomaterials degradation and immunogenicity. Moreover, the physiological environment within the body is very complex and unpredictable. Therefore, the practical or clinical application of biomaterials in PTT and its combination therapy strategies is a tremendous challenge.

Moreover, regulating TME to achieve better tumor treatment effects by PTT is another vital issue, even comprehensively mobilizing the advantages of materials to eliminate tumor cells as well as reduce side effects. The long-term metabolic rate and biocompatibility should also be considered in vivo while improving the targeting ability of nanoscale photosensitizers. For example, current biomaterials are generally restricted by the efficacy range of hyperthermia therapy and whether this deficiency will be avoided by regulating the TME. However, owing to the influence of skin and tissue thickness on NIR penetration efficiency, the regulation of NIR power and density also needs to be investigated. Specifically, the balance between toxicity, photothermal effects, and the promotion of osteogenesis is of primary concern.

Collectively, this review summarizes the currently available advanced biomaterials and combination therapeutic strategies applied in the PTT of malignant bone tumors. In particular, the design of multifunctional biomaterials should consider the advantages of PTT-related combination therapy, ensuring it is more appropriate for suppressing and rehabilitating malignant bone tumors. This combination strategy provides novel options for the specific combination therapy of malignant bone tumors in the coming years.

## Data Availability

Not applicable.

## Abbreviations

PTT: Photothermal therapy
TME: Tumor microenvironments
HSP: Heat shock protein
NIR: Near-infrared radiation
GO: Graphene oxide
BP: Black phosphorus
BCs: Bioceramic
PEEK: Polyether ether ketone
PDT: Photodynamic therapy
CDT: Chemodynamic therapy
GNPs: Gold nanoparticles
MOFs: Metal–organic frameworks
MSCs: Mesenchymal stem cells
ICG: Indocyanine green
Asp8: Eight aspartic oligopeptides
HA: Hydroxyapatite
BTTP: Bone tumor-targeting peptide
MMP: Matrix metalloproteinases
CPT: Chemo-photothermal therapy
CS: Chitosan
PDA: Polydopamine
SLN: Silica nanoparticles
CM: Curcumin
SCM: Stem cell membrane
ALN: Alendronate sodium
RGD: Tripeptide Arg-Gly-Asp
BSA: Bovine serum albumin
BC: Bioceramics
MSN: Mesoporous silica nanoparticles
rBMSC: Rabbit bone mesenchymal stem cells
NS: Nanosheets
NIR-I: The first near-infrared window
NIR-II: The second near-infrared window
BCN: Borocarnitride
PDLLA: Poly (D, L-Lactide)
CA: Carbon aerogel
AKT: Akermanite
ALG: Alginate
GelMA: Methacrylate gelatin
CPC: Calcium phosphate bone cement
PCL: Polycaprolactone
LDDS: Local drug delivery systems
ROS: Reactive oxygen species
H2O2: Hydrogen peroxide
Mn: Manganese
Bi: Bismuth
NO: Nitric oxide
RNS: Reactive nitrogen species
Nb2C: Niobium carbide
PD-L1: Anti-programmed cell death protein-1 ligand
PEI: Polyethyleneimine
DC: Dendritic cell
iDC: Intelligent organic dendritic cell
TAM: Tumor-associated macrophages
PBA: Prussian blue analog
CSCs: Cancer stem cells
CNH: Carbon nano angle
TDT: Thermodynamic therapy
GSH: Glutathione
GSSG: Glutathione disulfide
CNH: Carbon nanohorn
TRPV2: Transient receptor potential vanilloid family type 2

## References

[1] Ma HS, Jiang CA, Zhai D, Luo YX, Chen Y, Lv F (2016). A bifunctional biomaterial with photothermal effect fortumor therapy and bone regeNeration. Adv Funct Mater.

[2] Gosling T, Probst C, Langer F, Rosenthal H, Brunnemer U, Krettek C (2010). Diagnostics and treatment of primary bone tumors. Chirurg.

[3] Gao X, Li L, Cai X, Huang Q, Xiao J, Cheng Y (2021). Targeting nanoparticles for diagnosis and therapy of bone tumors: Opportunities and challenges. Biomaterials.

[4] Ban J, Fock V, Aryee DNT, Kovar H (2021). Mechanisms, diagnosis and treatment of bone metastases. Cells.

[5] Liao JF, Han RX, Wu YZ, Qian ZY (2021). Review of a new bone tumor therapy strategy based on bifunctional biomaterials. Bone Res.

[6] Yang Z, Zhao FJ, Zhang W, Yang ZY, Luo M, Liu L (2021). Degradable photothermal bioactive glass composite hydrogel for the sequential treatment of tumor-related bone defects: From anti-tumor to repairing bone defects. Chem Eng J.

[7] Fornetti J, Welm AL, Stewart SA (2018). Understanding the bone in cancer metastasis. J Bone Miner Res.

[8] Epstein-Peterson ZD, Sullivan A, Krishnan M, Chen JT, Ferrone M, Ready J (2015). Postoperative radiation therapy for osseous metastasis: Outcomes and predictors of local failure. Pract Radiat Oncol.

[9] Krishnan CK, Kim HS, Yun JY, Cho HS, Park JW, Han I (2018). Factors associated with local recurrence after surgery for bone metastasis to the extremities. J Surg Oncol.

[10] Saraf AJ, Fenger JM, Roberts RD (2018). Osteosarcoma: accelerating progress makes for a hopeful future. Front. Oncol..

[11] Berner K, Johannesen TB, Berner A, Haugland HK, Bjerkehagen B, Bohler PJ (2015). Time-trends on incidence and survival in a nationwide and unselected cohort of patients with skeletal osteosarcoma. Acta Oncol.

[12] Marchandet L, Lallier M, Charrier C, Baudhuin M, Ory B, Lamoureux F (2021). Mechanisms of resistance to conventional therapies for osteosarcoma. Cancers.

[13] Zhang Y, Liu X, Geng C, Shen H, Zhang Q, Miao Y (2023). Two hawks with one arrow: a review on bifunctional scaffolds for photothermal therapy and bone regeneration. Nanomaterials.

[14] Chatterjee DK, Diagaradjane P, Krishnan S (2011). Nanoparticle-mediated hyperthermia in cancer therapy. Ther Delivery.

[15] Chen G, Cao Y, Tang Y, Yang X, Liu Y, Huang D (2020). Advanced near-infrared light for monitoring and modulating the spatiotemporal dynamics of cell functions in living systems. Adv Sci.

[16] Li J, Yu X, Jiang Y, He S, Zhang Y, Luo Y (2021). Second near-infrared photothermal semiconducting polymer nanoadjuvant for enhanced cancer immunotherapy. Adv Mater.

[17] Ding X, Liow CH, Zhang M, Huang R, Li C, Shen H (2014). Surface plasmon resonance enhanced light absorption and photothermal therapy in the second near-infrared window. J Am Chem Soc.

[18] Yang Y, Fan X, Li L, Yang Y, Nuernisha A, Xue D, Huang W (2020). Semiconducting polymer nanoparticles as theranostic system for near-infrared-ii fluorescence imaging and photothermal therapy under safe laser fluence. ACS Nano.

[19] Kargozar S, Mozafari M, Ghodrat S, Fiume E, Baino F (2021). Copper-containing bioactive glasses and glass-ceramics: from tissue regeneration to cancer therapeutic strategies. Mater Sci Eng C Mater Biol Appl.

[20] Zhou M, Xing Y, Li X, Du X, Xu T, Zhang X (2020). Cancer cell membrane camouflaged semi-Yolk@Spiky-shell nanomotor for enhanced cell adhesion and synergistic therapy. Small.

[21] Malekzadeh R, Mortezazadeh T, Abdulsahib WK, Abdollah BB, Hamblin MR, Mansoori B (2023). Nanoarchitecture-based photothermal ablation of cancer: a systematic review. Entomol Res.

[22] Jiang Z, Li J, Chen S, Guo Q, Jing Z, Huang B (2020). Zoledronate and SPIO dual-targeting nanoparticles loaded with ICG for photothermal therapy of breast cancer tibial metastasis. Sci Rep.

[23] Au L, Zheng D, Zhou F, Li ZY, Li X, Xia Y (2008). A quantitative study on the photothermal effect of immuno gold nanocages targeted to breast cancer cells. ACS Nano.

[24] Huang X, Neretina S, El-Sayed MA (2009). Gold nanorods: From synthesis and properties to biological and biomedical applications. Adv Mater.

[25] Shi D, Cho HS, Chen Y, Xu H, Gu H, Lian J (2009). Fluorescent polystyrene-Fe3O4 composite nanospheres for in vivo imaging and hyperthermia. Adv Mater.

[26] Zhu CH, Lu Y, Chen JF, Yu SH (2014). Photothermal poly(N-isopropylacrylamide)/Fe3O4 nanocomposite hydrogel as a movable position heating source under remote control. Small.

[27] Markovic ZM, Harhaji-Trajkovic LM, Todorovic-Markovic BM, Kepić DP, Arsikin KM, Jovanović SP (2011). In vitro comparison of the photothermal anticancer activity of graphene nanoparticles and carbon nanotubes. Biomaterials.

[28] Wang L, Shi J, Zhang H, Li H, Gao Y, Wang Z, Wang H, Li L, Zhang C, Chen C, Zhang Z, Zhang Y (2013). Synergistic anticancer effect of RNAi and photothermal therapy mediated by functionalized single-walled carbon nanotubes. Biomaterials.

[29] Li JL, Bao HC, Hou XL, Sun L, Wang XG, Gu M (2012). Graphene oxide nanoparticles as a nonbleaching optical probe for two-photon luminescence imaging and cell therapy. Angew Chem Int Ed.

[30] Yang K, Hu L, Ma X, Ye S, Cheng L, Shi X (2012). Multimodal imaging guided photothermal therapy using functionalized graphene nanosheets anchored with magnetic nanoparticles. Adv Mater.

[31] Chen W, Ouyang J, Liu H, Chen M, Zeng K, Sheng J, et al. Black phosphorus nanosheet-based drug delivery system for synergistic photodynamic/photothermal/chemotherapy of cancer. Adv Mater. 2017;29(5):1603864. 10.1002/adma.201603864 27882622

[32] Zhao LY, Liu YM, Chang R, Xing RR, Yan XH (2019). Supramolecular photothermal nanomaterials as an emerging paradigm toward precision cancer therapy. Adv Funct Mater.

[33] Li L, Han X, Wang M, Li C, Jia T, Zhao X (2021). Recent advances in the development of near-infrared organic photothermal agents. Chem Eng J.

[34] Xu X, Chen X, Wang H, Mei X, Chen B, Li R (2022). Balancing the toxicity, photothermal effect, and promotion of osteogenesis: Photothermal scaffolds for malignant bone tumor therapy. Mater Today Adv.

[35] Lagos KJ, Buzza HH, Bagnato VS, Romero MP (2022). Carbon-based materials in photodynamic and photothermal therapies applied to tumor destruction. Int J Mol Sci.

[36] Maleki A, He J, Bochani S, Nosrati V, Shahbazi M-A, Guo B (2021). Multifunctional photoactive hydrogels for wound healing acceleration. ACS Nano.

[37] Sun W, Zhao X, Fan J, Du J, Peng X (2019). Boron dipyrromethene nano-photosensitizers for anticancer phototherapies. Small.

[38] Liao JF, Shi K, Jia YP, Wu YT, Qian ZY (2021). Gold nanorods and nanohydroxyapatite hybrid hydrogel for preventing bone tumor recurrence via postoperative photothermal therapy and bone regeneration promotion. Bioact Mater.

[39] Deng X, Shao Z, Zhao Y (2021). Solutions to the drawbacks of photothermal and photodynamic cancer therapy. Adv Sci.

[40] Dang W, Ma B, Li B, Huan Z, Ma N, Zhu H (2020). 3D printing of metal-organic framework nanosheets-structured scaffolds with tumor therapy and bone construction. Biofabrication.

[41] Fang Z, Chen J, Pan J, Liu G, Zhao C (2021). The development tendency of 3D-printed bioceramic scaffolds for applications ranging from bone tissue regeneration to bone tumor therapy. Front Bioeng Biotechnol.

[42] Cao YF, Liu MS, Cheng J, Yin JJ, Huang CS, Cui HY (2020). Acidity-triggered tumor-targeted nanosystem for synergistic therapy via a cascade of ROS generation and no release. ACS Appl Mater Interfaces.

[43] Zeng LL, Huang K, Wan YL, Zhang J, Yao XK, Jiang C (2020). Programmable starving-photodynamic synergistic cancer therapy. Sci China-Mat.

[44] Zhou X, Yan N, Cornel EJ, Cai HD, Xue SB, Xi H (2021). Bone-targeting polymer vesicles for simultaneous imaging and effective malignant bone tumor treatment. Biomaterials.

[45] Cheng H, Chawla A, Yang Y, Li Y, Zhang J, Jang HL, Khademhosseini A (2017). Development of nanomaterials for bone-targeted drug delivery. Drug Discov Today.

[46] Kermanizadeh A, Jacobsen NR, Murphy F, Powell L, Parry L, Zhang H, Moller P (2021). A review of the current state of nanomedicines for targeting and treatment of cancers: achievements and future challenges. Adv Ther.

[47] Wu JZ, Ghopry SA, Liu B, Shultz A (2023). Metallic and non-metallic plasmonic nanostructures for LSPR sensors. Micromachines.

[48] Kayani Z, Islami N, Behzadpour N, Zahraie N, Imanlou S, Tamaddon P, Mohammadi S (2021). Combating cancer by utilizing noble metallic nanostructures in combination with laser photothermal and X-ray radiotherapy. J Drug Deliv Sci Technol.

[49] Espinosa A, Kolosnjaj-Tabi J, Abou-Hassan A, Plan Sangnier A, Curcio A, Silva KA (2018). Magnetic (Hyper)Thermia or Photothermia? progressive comparison of iron oxide and gold nanoparticles heating in water, in cells, and in vivo. Adv Funct Mater.

[50] Gupta N, Malviya R (2021). Understanding and advancement in gold nanoparticle targeted photothermal therapy of cancer. Biochimica Et Biophysica Acta-Rev Cancer.

[51] Jain PK, Lee KS, El-Sayed IH, El-Sayed MA (2006). Calculated absorption and scattering properties of gold nanoparticles of different size, shape, and composition: applications in biological imaging and biomedicine. J Phys Chem B.

[52] Huang Y-C, Lei KF, Liaw J-W, Tsai S-W (2019). The influence of laser intensity activated plasmonic gold nanoparticle-generated photothermal effects on cellular morphology and viability: a real-time, long-term tracking and monitoring system. Photochem Photobiol Sci.

[53] Simon M, Jorgensen JT, Melander F, Andresen TL, Christensen A, Kjaer A (2021). Photothermal therapy as adjuvant to surgery in an orthotopic mouse model of human fibrosarcoma. Cancers.

[54] Yan Y, Gao X, Zhang S, Wang Y, Zhou Z, Xiao J (2019). A carboxyl-terminated dendrimer enables osteolytic lesion targeting and photothermal ablation of malignant bone tumors. ACS Appl Mater Interfaces.

[55] Ma Y, Chen L, Li X, Hu A, Wang H, Zhou H (2021). Rationally integrating peptide-induced targeting and multimodal therapies in a dual-shell theranostic platform for orthotopic metastatic spinal tumors. Biomaterials.

[56] Hofbauer LC, Bozec A, Rauner M, Jakob F, Perner S, Pantel K (2021). Novel approaches to target the microenvironment of bone metastasis. Nat Rev Clin Oncol.

[57] Schroeder A, Heller DA, Winslow MM, Dahlman JE, Pratt GW, Langer R (2012). Treating metastatic cancer with nanotechnology. Nat Rev Cancer.

[58] Gao X, Li L, Cai X, Huang Q, Xiao J, Cheng Y (2021). Targeting nanoparticles for diagnosis and therapy of bone tumors: opportunities and challenges. Biomaterials.

[59] Fang RH, Kroll AV, Gao W, Zhang L (2018). Cell membrane coating nanotechnology. Adv Mater.

[60] Song Y, Qu Z, Li J, Shi L, Zhao W, Wang H (2021). Fabrication of the biomimetic DOX/Au@Pt nanoparticles hybrid nanostructures for the combinational chemo/photothermal cancer therapy. J Alloys Compd.

[61] Zhang L, Ma X, Zhou W, Wu Q, Yan J, Xu X (2022). Combination of photothermal, prodrug and tumor cell camouflage technologies for triple-negative breast cancer treatment. Mater Today Adv.

[62] Kim MW, Lee G, Niidome T, Komohara Y, Lee R, Park YI (2020). Platelet-like gold nanostars for cancer therapy: the ability to treat cancer and evade immune reactions. Front Bioeng Biotechnol.

[63] Yang L, Hu YM, Liu YF, Liu YY, Miao S, Li Z (2020). Preparation and in vitro evaluation of doxorubicin loaded alendronate modified hollow gold nanoparticles for bone -targeted chemo-photothermal therapy. Mater Expr.

[64] Shen YP, Zou Y, Bie BL, Dong CJ, Lv YG (2023). Combining dual-targeted liquid metal nanoparticles with autophagy activation and mild photothermal therapy to treat metastatic breast cancer and inhibit bone destruction. Acta Biomater.

[65] Alvisi N, de Vries R (2023). Biomedical applications of solid-binding peptides and proteins. Materials today Bio.

[66] Tian J, Gu Y, Li Y, Liu T (2020). CD271 antibody-functionalized HGNs for targeted photothermal therapy of osteosarcoma stem cells. Nanotechnology.

[67] Liang S, Sun M, Lu Y, Shi S, Yang Y, Lin Y (2020). Cytokine-induced killer cells-assisted tumor-targeting delivery of Her-2 monoclonal antibody-conjugated gold nanostars with NIR photosensitizer for enhanced therapy of cancer. J Mater Chem B.

[68] Wang B, Huang Y. Antitumor effects of targeted killing of tumor-associated macrophages under photothermal conditions. Lasers Med Sci. 2021;37(1):299–307. 10.1007/s10103-021-03248-7 33439377

[69] Xiong SR, Xiong GS, Li ZH, Jiang Q, Yin J, Yin T (2021). Gold nanoparticle-based nanoprobes with enhanced tumor targeting and photothermal/photodynamic response for therapy of osteosarcoma. Nanotechnology.

[70] Wang Y, Yang J, Liu H, Wang X, Zhou Z, Huang Q, Song D, Cai X, Li L, Lin K, Xiao J, Liu P, Zhang Q, Cheng Y (2017). Osteotropic peptide-mediated bone targeting for photothermal treatment of bone tumors. Biomaterials.

[71] Yang C, Huang W, Gao Y, Liu Z, An N, Mu W (2020). Phototherapy ablation of rabbit orthotopic tumors by non-stoichiometric BiPO4-x nanoparticles. Chem Eng J.

[72] Lu J-W, Yang F, Ke Q-F, Xie X-T, Guo Y-P (2018). Magnetic nanoparticles modified-porous scaffolds for bone regeneration and photothermal therapy against tumors. Nanomed Nanotechnol Biol Med.

[73] Jie S, Guo X, Ouyang Z (2019). Tumor ablation using novel photothermal NaxWO3 nanoparticles against breast cancer osteolytic bone metastasis. Int J Nanomed.

[74] Zhao P-P, Ge Y-W, Liu X-L, Ke Q-F, Zhang J-W, Zhu Z-A (2020). Ordered arrangement of hydrated GdPO4 nanorods in magnetic chitosan matrix promotes tumor photothermal therapy and bone regeneration against breast cancer bone metastases. Chem Eng J.

[75] Nicolas-Boluda A, Laurent G, Bazzi R, Roux S, Donnadieu E, Gazeau F (2021). Two step promotion of a hot tumor immune environment by gold decorated iron oxide nanoflowers and light-triggered mild hyperthermia. Nanoscale.

[76] Chen T, Cen D, Ren Z, Wang Y, Cai X, Huang J (2019). Bismuth embedded silica nanoparticles loaded with autophagy suppressant to promote photothermal therapy. Biomaterials.

[77] Zhang Y, Sha R, Zhang L, Zhang W, Jin P, Xu W (2018). Harnessing copper-palladium alloy tetrapod nanoparticle-induced pro-survival autophagy for optimized photothermal therapy of drug-resistant cancer. Nat Commun.

[78] Ma Y, Jiang L, Hu J, Yuan Y (2022). Engineering a multiscale multifunctional theragenerative system for enhancing osteosarcoma therapy, bone regeneration and bacterial eradication. Chem Eng J.

[79] Ma YL, Jiang L, Hu J, Zhu EJ, Zhang N (2022). Developing a versatile multiscale therapeutic platform for osteosarcoma synergistic photothermo-chemotherapy with effective osteogenicity and antibacterial capability. ACS Appl Mater Interfaces.

[80] Zou Y, Chen X, Yang P, Liang G, Yang Y, Gu Z (2020). Regulating the absorption spectrum of polydopamine. Sci Adv.

[81] Li J, Zhang W, Ji W, Wang J, Wang N, Wu W (2021). Near infrared photothermal conversion materials: mechanism, preparation, and photothermal cancer therapy applications. J Mater Chem B.

[82] Jin AT, Wang YT, Lin KL, Jiang L (2020). Nanoparticles modified by polydopamine: working as "drug" carriers. Bioact Mater.

[83] Zhen X, Pu K, Jiang X (2021). Photoacoustic imaging and photothermal therapy of semiconducting polymer nanoparticles: signal amplification and second near-infrared construction. Small.

[84] Son J, Yi G, Yoo J, Park C, Koo H, Choi HS (2019). Light-responsive nanomedicine for biophotonic imaging and targeted therapy. Adv Drug Deliv Rev.

[85] Xue Y, Niu W, Wang M, Chen M, Guo Y, Lei B (2020). Engineering a biodegradable multifunctional antibacterial bioactive nanosystem for enhancing tumor photothermo- chemotherapy and bone regeneration. ACS Nano.

[86] Quoc-Viet L, Suh J, Choi JJ, Park GT, Lee JW, Shim G (2019). In Situ nanoadjuvant-assembled tumor vaccine for preventing long-term recurrence. ACS Nano.

[87] An P, Fan F, Gu D, Gao Z, Hossain AMS, Sun B (2020). Photothermal-reinforced and glutathione-triggered in Situ cascaded nanocatalytic therapy. J Contr Rel.

[88] Guo H, Xia Y, Feng K, Qu X, Zhang C, Wan F (2020). Surface engineering of metal-organic framework as pH-/NIR-responsive nanocarrier for imaging-guided chemo-photothermal therapy. Int J Nanomed.

[89] Sun X, Meng Z, Yu Q, Wang X, Zhao Z (2021). Engineering PDA-coated CM-CS nanoparticles for photothermo-chemotherapy of osteosarcoma and bone regeneration. Biochem Eng J.

[90] Zhang W, Huang X (2022). Stem cell membrane-camouflaged targeted delivery system in tumor. Mater Today Bio.

[91] Zhang M, Zhang F, Liu T, Shao P, Duan L, Yan J, Mu X (2020). Polydopamine nanoparticles camouflaged by stem cell membranes for synergistic chemo-photothermal therapy of malignant bone tumors. Int J Nanomed.

[92] Liu W-B, Dong S-H, Hu W-H, Gao M, Li T, Ji Q-B (2022). A simple, universal and multifunctional template agent for personalized treatment of bone tumors. Bioact Mater.

[93] Gruenherz L, Prein C, Winkler T, Kirsch M, Hopfner U, Streichert T (2020). Osteoidosis leads to altered differentiation and function of osteoclasts. J Cell Mol Med.

[94] Kong YL, Zhou L, Liao SY, Wang CP, Chen J, Cai XP (2022). Dual peptide-engineered and gadolinium-doped polydopamine particles as targeted nanotheranostics for the treatment of osteosarcoma and related osteolysis. Chem Eng J.

[95] Wang YT, Cui JJ, Chen JJ, Wan JY, Liang YK, Qi M (2022). Novel bone tumor cell targeting nanosystem for chemo-photothermal therapy of malignant bone tumors. Chem Eng J.

[96] Wei Y, Gao L, Wang L, Shi L, Wei ED, Zhou BT (2017). Polydopamine and peptide decorated doxorubicin-loaded mesoporous silica nanoparticles as a targeted drug delivery system for bladder cancer therapy. Drug Deliv.

[97] Wu M, Meng Q, Chen Y, Zhang L, Li M, Cai X (2016). Large pore-sized hollow mesoporous organosilica for redox-responsive gene delivery and synergistic cancer chemotherapy. Adv Mater.

[98] Ong C, Cha BG, Kim J (2019). Mesoporous silica nanoparticles doped with gold nanoparticles for combined cancer immunotherapy and photothermal therapy. ACS Appl Bio Mater.

[99] Zhang J, Miao Y, Ni W, Xiao H, Zhang J (2019). Cancer cell membrane coated silica nanoparticles loaded with ICG for tumour specific photothermal therapy of osteosarcoma. Artificial Cells, Nanomed Biotechnol.

[100] Croissant JG, Fatieiev Y, Khashab NM (2017). Degradability and clearance of silicon, organosilica, silsesquioxane, silica mixed oxide, and mesoporous silica nanoparticles. Adv Mater.

[101] Huang P, Chen Y, Lin H, Yu L, Zhang L, Wang L (2017). Molecularly organic/inorganic hybrid hollow mesoporous organosilica nanocapsules with tumor-specific biodegradability and enhanced chemotherapeutic functionality. Biomaterials.

[102] Li D, Zhang T, Min C, Huang H, Tan D, Gu W (2020). Biodegradable theranostic nanoplatforms of albumin-biomineralized nanocomposites modified hollow mesoporous organosilica for photoacoustic imaging guided tumor synergistic therapy. Chem Eng J.

[103] Joguparthi V, Xiang T-X, Anderson BD (2008). Liposome transport of hydrophobic drugs: Gel phase lipid bilayer permeability and partitioning of the lactone form of a hydrophobic camptothecin, DB-67. J Pharm Sci.

[104] Demirbolat GM, Erdogan O, Coskun GP, Cevik O (2022). PEG4000 modified liposomes enhance the solubility of quercetin and improve the liposome functionality: in vitro characterization and the cellular efficacy. Turk J Chem.

[105] Li L, Yang S, Song L, Zeng Y, He T, Wang N (2018). An endogenous vaccine based on fluorophores and multivalent immunoadjuvants regulates tumor micro-environment for synergistic photothermal and immunotherapy. Theranostics.

[106] Chen Y, Chen Q, Zhu Q, Liu J, Li Y, Gao X (2019). Small molecular theranostic assemblies functionalized by doxorubicin-hyaluronic acid-methotrexate prodrug for multiple tumor targeting and imaging-guided combined chemo-photothermal therapy. Mol Pharm.

[107] Suleman A, Kondiah PPD, Mabrouk M, Choonara YE (2021). The application of 3D-printing and nanotechnology for the targeted treatment of osteosarcoma. Front Mater.

[108] Tang G, Liu Z, Liu Y, Yu J, Wang X, Tan Z (2021). Recent trends in the development of bone regenerative biomaterials. Front Cell Dev Biol.

[109] Dong S, Wang X, Shen Steve G, Wang X, Lin K (2020). Research progress on functional modifications and applications of bioceramic scaffolds. J Inorg Mater.

[110] Sharifi E, Bigham A, Yousefiasl S, Trovato M, Ghomi M, Esmaeili Y (2022). Mesoporous bioactive glasses in cancer diagnosis and therapy: stimuli-responsive, toxicity, immunogenicity, and clinical translation. Adv Sci.

[111] Ma H, Feng C, Chang J, Wu C (2018). 3D-printed bioceramic scaffolds: From bone tissue engineering to tumor therapy. Acta Biomater.

[112] Srinath P, Abdul Azeem P, Venugopal Reddy K (2020). Review on calcium silicate-based bioceramics in bone tissue engineering. Int J Appl Ceram Technol.

[113] Ma H, Ma Z, Chen Q, Li W, Liu X, Ma X (2020). Bifunctional, copper-doped, mesoporous silica nanosphere-modified, bioceramic scaffolds for bone tumor therapy. Front Chem.

[114] de Melo-Diogo D, Lima-Sousa R, Alves CG, Costa EC, Louro RO, Correia IJ (2018). Functionalization of graphene family nanomaterials for application in cancer therapy. Colloids Surf B Biointerfaces.

[115] Luong-Van EK, Madanagopal TT, Rosa V (2020). Mechanisms of graphene influence on cell differentiation. Mater Today Chem.

[116] Shen A, Zhao C, Mao L, Wu Y, Zhao B, Lin K (2020). Adhesive graphene grown on bioceramics with photothermal property. Mater Today Chem.

[117] Du J, Ding H, Fu S, Li D, Yu B (2022). Bismuth-coated 80S15C bioactive glass scaffolds for photothermal antitumor therapy and bone regeneration. Front Bioeng Biotechnol.

[118] Ma H, Luo J, Sun Z, Xia L, Shi M, Liu M (2016). 3D printing of biomaterials with mussel-inspired nanostructures for tumor therapy and tissue regeneration. Biomaterials.

[119] Li B, Wang X, Chen L, Zhou Y, Dang W, Chang J (2018). Ultrathin Cu-TCPP MOF nanosheets: a new theragnostic nanoplatform with magnetic resonance/near-infrared thermal imaging for synergistic phototherapy of cancers. Theranostics.

[120] Zhang X, Li YZ, Dong XM, Wang H, Chen B, Li RY (2022). 3D-printed bioactive ceramic scaffolds with MoSe2 nanocrystals as photothermal agents for bone tumor therapy. RSC Adv.

[121] Rasool K, Helal M, Ali A, Ren CE, Gogotsi Y, Mahmoud KA (2016). Antibacterial activity of Ti3C2Tx MXene. ACS Nano.

[122] Yang BW, Chen Y, Shi JL (2018). Material chemistry of two-dimensional inorganic nanosheets in cancer theranostics. Chem.

[123] Pan S, Yin J, Yu L, Zhang C, Zhu Y, Gao Y (2020). 2D MXene-integrated 3D-printing scaffolds for augmented osteosarcoma phototherapy and accelerated tissue reconstruction. Adv Sci.

[124] Wu JYZ, You LY, Lan L, Lee HJ, Chaudhry ST, Li R (2017). Semiconducting polymer nanoparticles for centimeters-deep photoacoustic imaging in the second near-infrared window. Adv Mat.

[125] Yin J, Pan S, Guo X, Gao Y, Zhu D, Yang Q (2021). Nb2C MXene-functionalized scaffolds enables osteosarcoma phototherapy and angiogenesis/osteogenesis of bone defects. Nano-Micro Lett.

[126] Zhao C, Shen A, Zhang L, Lin K, Wang X (2020). Borocarbonitrides nanosheets engineered 3D-printed scaffolds for integrated strategy of osteosarcoma therapy and bone regeneration. Chem Eng J.

[127] Martin C, Kostarelos K, Prato M, Bianco A (2019). Biocompatibility and biodegradability of 2D materials: graphene and beyond. Chem Commun.

[128] Dang WT, Ma B, Li B, Huan ZG, Ma N, Zhu HB, Wu C (2020). 3D printing of metal-organic framework nanosheets-structured scaffolds with tumor therapy and bone construction. Biofabrication.

[129] Dang W, Yi K, Ju E, Jin Y, Xu Y, Wang H (2021). 3D printed bioceramic scaffolds as a universal therapeutic platform for synergistic therapy of osteosarcoma. ACS Appl Mater Interfaces.

[130] Dang W, Jin Y, Yi K, Ju E, Zhuo C, Wei H (2021). Hemin particles-functionalized 3D printed scaffolds for combined photothermal and chemotherapy of osteosarcoma. Chem Eng J.

[131] Dang W, Ma B, Huan Z, Lin R, Wang X, Li T (2019). LaB6 surface chemistry-reinforced scaffolds for treating bone tumors and bone defects. Appl Mater Today.

[132] Dong S, Zhang Y-N, Wang J, Cui R, Yu X, Zhao G (2020). A novel multifunctional carbon aerogel-coated platform for osteosarcoma therapy and enhanced bone regeneration. J Mater Chem B.

[133] Ma H, Li T, Huan Z, Zhang M, Yang Z, Wang J (2018). 3D printing of high-strength bioscaffolds for the synergistic treatment of bone cancer. NPG Asia Mater.

[134] Yao M, Zou Q, Zou W, Xie Z, Li Z, Zhao X, Du C (2021). Bifunctional scaffolds of hydroxyapatite/poly(dopamine)/carboxymethyl chitosan with osteogenesis and anti-osteosarcoma effect. Biomater Sci.

[135] Zhang X, Ma J (2021). Photothermal effect of 3D printed hydroxyapatite composite scaffolds incorporated with graphene nanoplatelets. Ceram Int.

[136] Wang X, Zhai D, Yao X, Wang Y, Ma H, Yu X, Du L, Lin H, Wu C (2022). 3D printing of pink bioceramic scaffolds for bone tumor tissue therapy. Appl Mat Today.

[137] Saber-Samandari S, Mohammadi-Aghdam M, Saber-Samandari S (2019). A novel magnetic bifunctional nanocomposite scaffold for photothermal therapy and tissue engineering. Int J Biol Macromol.

[138] Zhuang H, Lin R, Liu Y, Zhang M, Zhai D, Huan Z (2019). Three-dimensional-printed bioceramic scaffolds with osteogenic activity for simultaneous photo/magnetothermal therapy of bone tumors. ACS Biomater Sci Eng.

[139] Du X, Yu B, Pei P, Ding H, Yu B, Zhu Y (2018). 3D printing of pearl/CaSO4 composite scaffolds for bone regeneration. J Mater Chem B.

[140] Pei P, Qi X, Du X, Zhu M, Zhao S, Zhu Y (2016). Three-dimensional printing of tricalcium silicate/mesoporous bioactive glass cement scaffolds for bone regeneration. J Mater Chem B.

[141] Fu S, Hu H, Chen J, Zhu Y, Zhao S (2020). Silicone resin derived larnite/C scaffolds via 3D printing for potential tumor therapy and bone regeneration. Chem Eng J.

[142] Feng Q, Lin S, Zhang KY, Dong CQ, Wu TY, Huang HQ (2017). Sulfated hyaluronic acid hydrogels with retarded degradation and enhanced growth factor retention promote hMSC chondrogenesis and articular cartilage integrity with reduced hypertrophy. Acta Biomater.

[143] Tan B, Huang L, Wu Y, Liao J (2021). Advances and trends of hydrogel therapy platform in localized tumor treatment: a review. J Biomed Mater Res Part A.

[144] Zhang X, Xia L-Y, Chen X, Chen Z, Wu F-G (2017). Hydrogel-based phototherapy for fighting cancer and bacterial infection. Sci China-Mat.

[145] Tan B, Wu Y, Wu Y, Shi K, Han R, Li Y (2021). Curcumin-Microsphere/IR820 hybrid bifunctional hydrogels for In Situ osteosarcoma chemo-co-thermal therapy and bone reconstruction. ACS Appl Mater Interfaces.

[146] Bacakova L, Pajorova J, Tomkova M, Matejka R, Broz A, Stepanovska J (2020). Applications of Nanocellulose/Nanocarbon composites: focus on biotechnology and medicine. Nanomaterials.

[147] Hou M, Liu W, Zhang L, Zhang L, Xu Z, Cao Y (2020). Responsive agarose hydrogel incorporated with natural humic acid and MnO2 nanoparticles for effective relief of tumor hypoxia and enhanced photo-induced tumor therapy. Biomater Sci.

[148] Luo S, Wu J, Jia Z, Tang P, Sheng J, Xie C (2019). An injectable, bifunctional hydrogel with photothermal effects for tumor therapy and bone regeneration. Macromol Biosci.

[149] Dutta SD, Hexiu J, Patel DK, Ganguly K, Lim KT (2021). 3D-printed bioactive and biodegradable hydrogel scaffolds of alginate/gelatin/cellulose nanocrystals for tissue engineering. Int J Biol Macromol.

[150] Grijalvo S, Nieto-Diaz M, Maza RM, Eritja R, Diaz DD (2019). Alginate hydrogels as scaffolds and delivery systems to repair the damaged spinal cord. Biotechnol J.

[151] Liu B, Gu X, Sun Q, Jiang S, Sun J, Liu K (2021). Injectable In Situ induced robust hydrogel for photothermal therapy and bone fracture repair. Adv Funct Mater.

[152] Kim J, Hope CM, Gantumur N, Perkins GB, Stead SO, Yue Z (2020). Encapsulation of human natural and induced regulatory T-cells in IL-2 and CCL1 supplemented alginate-GelMA hydrogel for 3D bioprinting. Adv Funct Mater.

[153] Shirahama H, Lee BH, Tan LP, Cho NJ (2016). Precise tuning of facile one-pot gelatin methacryloyl (GelMA) synthesis. Sci Rep.

[154] Wu YT, Zhang X, Tan BW, Shan Y, Zhao X, Liao JF (2022). Near-infrared light control of GelMA/PMMA/PDA hydrogel with mild photothermal therapy for skull regeneration. Biomater Adv.

[155] Miao Y, Shi X, Li Q, Hao L, Liu L, Liu X (2019). Engineering natural matrices with black phosphorus nanosheets to generate multi-functional therapeutic nanocomposite hydrogels. Biomater Sci.

[156] Liu XY, Zhang Y, Wu H, Tang JW, Zhou J, Zhao JL (2023). A conductive gelatin methacrylamide hydrogel for synergistic therapy of osteosarcoma and potential bone regeneration. Int J Biol Macromol.

[157] Mjoberg B, Pettersson H, Rosenqvist R, Rydholm A (1984). Bone cement, thermal injury and the radiolucent zone. Acta Orthop Scand.

[158] Xu C, Ma B, Peng J, Gao L, Xu Y, Huan Z, Chang J (2019). Tricalcium silicate/graphene oxide bone cement with photothermal properties for tumor ablation. J Mater Chem B.

[159] Qu Y, Zhuang H, Zhang M, Wang Y, Zhai D, Ma B (2021). Bone cements for therapy and regeneration for minimally invasive treatment of neoplastic bone defects. J Mater Chem B.

[160] Zhu C, He M, Mao L, Li T, Zhang L, Liu L (2021). Titanium-interlayer mediated hydroxyapatite coating on polyetheretherketone: a prospective study in patients with single-level cervical degenerative disc disease. J Transl Med.

[161] Kang K-T, Koh Y-G, Son J, Yeom JS, Park J-H, Kim H-J (2017). Biomechanical evaluation of pedicle screw fixation system in spinal adjacent levels using polyetheretherketone, carbon-fiber-reinforced polyetheretherketone, and traditional titanium as rod materials. Composites Part B.

[162] He MM, Zhu C, Xu H, Sun D, Chen C, Feng GJ (2020). Conducting polyetheretherketone nanocomposites with an electrophoretically deposited bioactive coating for bone tissue regeneration and multimodal therapeutic applications. ACS Appl Mater Interfaces.

[163] Zhu C, He MM, Sun D, Huang Y, Huang LZ, Du MX (2021). 3D-printed multifunctional polyetheretherketone bone scaffold for multimodal treatment of osteosarcoma and osteomyelitis. ACS Appl Mater Interfaces.

[164] He MM, Zhu C, Sun D, Liu Z, Du MX, Huang Y (2022). Layer-by-layer assembled black phosphorus/chitosan composite coating for multi-functional PEEK bone scaffold. Composites, Part B.

[165] Ma K, Liao C, Huang L, Liang R, Zhao J, Zheng L (2021). Electrospun PCL/MoS2 nanofiber membranes combined with NIR-triggered photothermal therapy to accelerate bone regeneration. Small.

[166] Xue X, Zhang H, Liu H, Wang S, Li J, Zhou Q (2022). Rational design of multifunctional CuS nanoparticle-PEG composite soft hydrogel-coated 3D hard polycaprolactone scaffolds for efficient bone regeneration. Adv Funct Mater.

[167] Yang C, Ma H, Wang Z, Younis MR, Liu C, Wu C (2021). 3D printed Wesselsite nanosheets functionalized scaffold facilitates NIR-II photothermal therapy and vascularized bone regeneration. Adv Sci.

[168] Shalapour S, Karin M (2019). Pas de Deux: control of anti-tumor immunity by cancer-associated inflammation. Immunity.

[169] Balkwill F, Charles KA, Mantovani A (2005). Smoldering and polarized inflammation in the initiation and promotion of malignant disease. Cancer Cell.

[170] Zeng WF, Li Z, Chen HZ, Zeng XW, Mei L (2022). An optimal portfolio of photothermal combined immunotherapy. Cell Rep Phys Sci.

[171] Bian W, Wang Y, Pan Z, Chen N, Li X, Wong W-L (2021). Review of functionalized nanomaterials for photothermal therapy of cancers. ACS Appl Nano Mater.

[172] McCabe-Lankford EE, Brown TL, Levi-Polyachenko NH (2018). Assessing fluorescence detection and effective photothermal therapy of near-infrared polymer nanoparticles using alginate tissue phantoms. Lasers Surg Med.

[173] Ting G, Zhang W, Fei G, Zhu L, Ping S, Li W (2022). A bone-targeting drug delivery vehicle of a metal-organic framework conjugate with zoledronate combined with photothermal therapy for tumor inhibition in cancer bone metastasis. Biomater Sci.

[174] Li W, Cao Z, Yu L, Huang Q, Zhu D, Lu C (2021). Hierarchical drug release designed Au @PDA-PEG-MTX NPs for targeted delivery to breast cancer with combined photothermal-chemotherapy. J Nanobiotechnol.

[175] Barroug A, Kuhn LT, Gerstenfeld LC, Glimcher MJ (2004). Interactions of cisplatin with calcium phosphate nanoparticles: in vitro controlled adsorption and release. J Orthop Res.

[176] Ferrari S, Serra M (2015). An update on chemotherapy for osteosarcoma. Exp Opin Pharmacother.

[177] Chen T, Li T, Wang J (2021). Nanoscale Au@SiO2-drug/VEGF as an in vivo probe for osteosarcoma diagnosis and therapy. Oncol Lett.

[178] Lu Y, Li L, Lin Z, Li M, Hu X, Zhang Y (2018). Enhancing osteosarcoma killing and CT imaging using ultrahigh drug loading and NIR-responsive Bismuth Sulfide@Mesoporous silica nanoparticles. Adv Healthc Mater.

[179] Shao H, Cheng S, Yao M, Ji X, Zhong H, Wang D (2021). A pH-response chemotherapy synergistic photothermal therapy for tumor suppression and bone regeneration by mussel-inspired Mg implant. Regener Biomater.

[180] Meng ZY, Liu YC, Xu KX, Sun X, Yu QW, Wu ZQ (2021). Biomimetic polydopamine-modified silk fibroin/curcumin nanofibrous scaffolds for chemo-photothermal therapy of bone tumor. ACS Omega.

[181] Riabenko TV, Korenkov OV, Dmytruk SM, Yarmolenko OS, Ponurko AA, Pernakov MS (2022). MORPHOLOGICAL FEATURES OF TUBULAR BONES REPARATIVE REGENERATION UNDER THE INFLUENCE OF ANTITUMOR CHEMOTHERAPEUTICS. Wiad Lek.

[182] Zhou L, Kuai F, Shi Q, Yang H (2020). Doxorubicin restrains osteogenesis and promotes osteoclastogenesis in vitro. Am J Transl Res.

[183] Li X, Yang Z, Fang L, Ma C, Zhao Y, Liu H (2021). Hydrogel composites with different dimensional nanoparticles for bone regeneration. Macromol Rapid Commun.

[184] Badila AE, Radulescu DM, Niculescu A-G, Grumezescu AM, Radulescu M, Radulescu AR (2021). Recent advances in the treatment of bone metastases and primary bone tumors: an up-to-date review. Cancers.

[185] Yang F, Lu J, Ke Q, Peng X, Guo Y, Xie X (2018). Magnetic mesoporous calcium sillicate/chitosan porous scaffolds for enhanced bone regeneration and photothermal-chemotherapy of osteosarcoma. Sci Rep.

[186] Wang YT, Huang Q, He X, Chen H, Zou Y, Li YW (2018). Multifunctional melanin-like nanoparticles for bone-targeted chemo-photothermal therapy of malignant bone tumors and osteolysis. Biomaterials.

[187] Wang C, Ye X, Zhao Y, Bai L, He Z, Tong Q (2020). Cryogenic 3D printing of porous scaffolds for in situ delivery of 2D black phosphorus nanosheets, doxorubicin hydrochloride and osteogenic peptide for treating tumor resection-induced bone defects. Biofabrication.

[188] Sun J, Xing F, Braun J, Traub F, Rommens PM, Xiang Z (2021). Progress of phototherapy applications in the treatment of bone cancer. Int J Mol Sci.

[189] Barnes KD, Shafirstein G, Webber JS, Koonce NA, Harris Z, Griffin RJ (2013). Hyperthermia-enhanced indocyanine green delivery for laser-induced thermal ablation of carcinomas. Int J Hyperthermia.

[190] Liu Y, Lin R, Ma L, Zhuang H, Feng C, Chang J (2020). Mesoporous bioactive glass for synergistic therapy of tumor and regeneration of bone tissue. Appl Mat Today.

[191] Xu Y, Shi Z, Zhang LE, Brown EMB, Wu A (2016). Layered bismuth oxyhalide nanomaterials for highly efficient tumor photodynamic therapy. Nanoscale.

[192] Cheng J, Wang W, Xu X, Lin Z, Xie C, Zhang Y (2020). AgBiS2 nanoparticles with synergistic photodynamic and bioimaging properties for enhanced malignant tumor phototherapy. Mat Sci Eng C-Materials Biol Appl.

[193] De Leeuw AAC, Van Vulpen M, Van de Kamer JB, Warlam-Rodenhuis CC, Lagendijk JJW (2003). Increasing the systemic temperature during regional hyperthermia: effect of a cooling strategy on tumour temperatures and side-effects. Int J Hyperthermia.

[194] Zhu J, Zou J, Zhang Z, Zhang J, Sun Y, Dong X (2019). An NIR triphenylamine grafted BODIPY derivative with high photothermal conversion efficiency and singlet oxygen generation for imaging guided phototherapy. Mater Chem Front.

[195] Wu Z, Tian Q, Wang J, Feng Y, Li L, Xu C (2022). A bone implant with NIR-responsiveness for eliminating osteosarcoma cells and promoting osteogenic differentiation of BMSCs. Colloids Surf B.

[196] Zhou W, Chen Y, Zhang Y, Xin X, Li R, Xie C (2020). Iodine-rich semiconducting polymer nanoparticles for CT/Fluorescence dual-modal imaging-guided enhanced photodynamic therapy. Small.

[197] Yuan Y, Diao S, Ni X, Zhang D, Yi W, Jian C (2022). Peptide-based semiconducting polymer nanoparticles for osteosarcoma-targeted NIR-II fluorescence/NIR-I photoacoustic dual-model imaging and photothermal/photodynamic therapies. J Nanobiotechnol.

[198] Fahey JM, Girotti AW (2015). Accelerated migration and invasion of prostate cancer cells after a photodynamic therapy-like challenge: Role of nitric oxide. Nitric Oxide.

[199] Deng YY, Jia F, Chen SY, Shen ZD, Jin Q, Fu GS (2018). Nitric oxide as an all-rounder for enhanced photodynamic therapy: Hypoxia relief, glutathione depletion and reactive nitrogen species generation. Biomaterials.

[200] Luo Z, Zhao Q, Liu J, Liao J, Peng R, Xi Y (2017). Fluorescent real-time quantitative measurements of intracellular peroxynitrite generation and inhibition. Anal Biochem.

[201] Lee J-H, Uyama H, Kwon O-K, Kim Y-J (2021). Nitric oxide and reactive oxygen species-releasing polylactic acid monolith for enhanced photothermal therapy of osteosarcoma. J Ind Eng Chem.

[202] Yang Q, Yin H, Xu T, Zhu D, Yin J, Chen Y (2020). Engineering 2D mesoporous Silica@MXene-Integrated 3D-printing scaffolds for combinatory osteosarcoma therapy and NO-augmented bone regeneration. Small.

[203] Zhang G, Wu Z, Yang Y, Shi J, Lv J, Fang Y (2022). A multifunctional antibacterial coating on bone implants for osteosarcoma therapy and enhanced osteointegration. Chem Eng J.

[204] Wan M, Chen H, Wang Q, Niu Q, Xu P, Yu Y (2019). Bio-inspired nitric-oxide-driven nanomotor. Nat Commun.

[205] Wang Y-C, Dai H-L, Li Z-H, Meng Z-Y, Xiao Y, Zhao Z (2021). Mesoporous polydopamine-coated hydroxyapatite nano-composites for ROS-triggered nitric oxide-enhanced photothermal therapy of osteosarcoma. J Mater Chem B.

[206] Bock FJ, Tait SWG (2020). Mitochondria as multifaceted regulators of cell death. Nat Rev Mol Cell Biol.

[207] Qi T, Chen B, Wang Z, Du H, Liu D, Yin Q (2019). A pH-Activatable nanoparticle for dual-stage precisely mitochondria-targeted photodynamic anticancer therapy. Biomaterials.

[208] Zeng W-N, Yu Q-P, Wang D, Liu J-L, Yang Q-J, Zhou Z-K (2021). Mitochondria-targeting graphene oxide nanocomposites for fluorescence imaging-guided synergistic phototherapy of drug-resistant osteosarcoma. J Nanobiotechnol.

[209] Schumacher TN, Schreiber RD (2015). Neoantigens in cancer immunotherapy. Science.

[210] Tseng JC, Granot T, DiGiacomo V, Levin B, Meruelo D (2010). Enhanced specific delivery and targeting of oncolytic Sindbis viral vectors by modulating vascular leakiness in tumor. Cancer Gene Ther.

[211] Song K-H, Oh SJ, Kim S, Cho H, Lee H-J, Song JS (2020). HSP90A inhibition promotes anti-tumor immunity by reversing multi-modal resistance and stem-like property of immune-refractory tumors. Nat Commun.

[212] Zhuang X, Zhang H, Li X, Li X, Cong M, Peng F (2017). Differential effects on lung and bone metastasis of breast cancer by Wnt signalling inhibitor DKK1. Nat Cell Biol.

[213] Yang C, Blum NT, Lin J, Qu J, Huang P (2020). Biomaterial scaffold-based local drug delivery systems for cancer immunotherapy. Sci Bull.

[214] Hou Y-J, Yang X-X, Liu R-Q, Zhao D, Guo C-X, Zhu A-C (2020). Pathological mechanism of photodynamic therapy and photothermal therapy based on nanoparticles. Int J Nanomed.

[215] He C, Yu L, Yao H, Chen Y, Hao Y (2021). Combinatorial photothermal 3D-printing scaffold and checkpoint blockade inhibits growth/metastasis of breast cancer to bone and accelerates osteogenesis. Adv Funct Mater.

[216] Zhao X, Han Y, Sun Y, Feng W, Liu J, Li D, Wang T (2021). Combining photothermal ablation-based vaccine with immune checkpoint blockade for synergistic osteosarcoma immunotherapy. Mater Des.

[217] Sun Z, Deng G, Peng X, Xu X, Liu L, Peng J (2021). Intelligent photothermal dendritic cells restart the cancer immunity cycle through enhanced immunogenic cell death. Biomaterials.

[218] Fu C, Peng P, Loschko J, Feng L, Phuong P, Cui W, Lee KP (2020). Plasmacytoid dendritic cells cross-prime naive CD8 T cells by transferring antigen to conventional dendritic cells through exosomes. Proc Natl Acad Sci USA.

[219] Liu KY, Liao YX, Zhou ZF, Zhang L, Jiang YY, Lu HL (2022). Photothermal-triggered immunogenic nanotherapeutics for optimizing osteosarcoma therapy by synergizing innate and adaptive immunity. Biomaterials.

[220] Valeta-Magara A, Gadi A, Volta V, Walters B, Arju R, Giashuddin S (2019). Inflammatory breast cancer promotes development of M2 tumor-associated macrophages and cancer mesenchymal cells through a complex chemokine network. Cancer Res.

[221] Zhou B, Yang Y, Li C (2019). SIRT1 inhibits hepatocellular carcinoma metastasis by promoting M1 macrophage polarization via NF-κB pathway. Onco Targets Ther.

[222] Deng X, Liang H, Yang W, Shao Z (2020). Polarization and function of tumor-associated macrophages mediate graphene oxide-induced photothermal cancer therapy. J Photochem Photobiol B.

[223] Sun X, Liu D, Xu X, Shen Y, Huang Y, Zeng Z (2020). NIR-triggered thermo-responsive biodegradable hydrogel with combination of photothermal and thermodynamic therapy for hypoxic tumor. Asian J Pharm Sci.

[224] Tang Z, Liu Y, He M, Bu W (2019). Chemodynamic Therapy: Tumour Microenvironment-Mediated Fenton and Fenton-like Reactions. Angew Chem Int Ed.

[225] Tang ZM, Zhang HL, Liu YY, Ni DL, Zhang H, Zhang JW (2017). Antiferromagnetic pyrite as the tumor microenvironment-mediated nanoplatform for self-enhanced tumor imaging and therapy. Adv Mater.

[226] Park KM, Baek K, Ko YH, Shrinidhi A, Murray J, Jang WH (2018). Mono-allyloxylated cucurbit 7 uril acts as an unconventional amphiphile to form light-responsive vesicles. Angew Chem Int Ed.

[227] Hao Y, Mao L, Zhang R, Liao X, Yuan M, Liao W (2021). Multifunctional biodegradable Prussian blue analogue for synergetic photothermal/photodynamic/chemodynamic therapy and intrinsic tumor metastasis inhibition. ACS Appl Bio Mater.

[228] Sun PF, Qu F, Zhang C, Cheng PF, Li XY, Shen QM (2022). NIR-II excitation phototheranostic platform for synergistic photothermal therapy/chemotherapy/chemodynamic therapy of breast cancer bone metastases. Adv Sci.

[229] Chen H, Fu Y, Feng K, Zhou Y, Wang X, Huang H (2021). Polydopamine-coated UiO-66 nanoparticles loaded with perfluorotributylamine/tirapazamine for hypoxia-activated osteosarcoma therapy. J Nanobiotechnol.

[230] Zhou Y, Fan S, Feng L, Huang X, Chen X (2021). Manipulating intratumoral fenton chemistry for enhanced chemodynamic and chemodynamic-synergized multimodal therapy. Adv Mater.

[231] Chu X, Zhang LF, Li YL, He Y, Zhang Y, Du C (2023). NIR responsive doxorubicin-loaded hollow copper ferrite @ polydopamine for synergistic chemodynamic/photothermal/chemo-therapy. Small.

[232] Phi LTH, Sari IN, Yang Y-G, Lee S-H, Jun N, Kim KS (2018). Cancer Stem Cells (CSCs) in drug resistance and their therapeutic implications in cancer treatment. Stem Cells Int.

[233] Yu Y, Yang X, Reghu S, Kaul SC, Wadhwa R, Miyako E (2020). Photothermogenetic inhibition of cancer stemness by near-infrared-light-activatable nanocomplexes. Nat Commun.

[234] Gao D, Chen T, Chen S, Ren X, Han Y, Li Y (2021). Targeting hypoxic tumors with hybrid nanobullets for oxygen-independent synergistic photothermal and thermodynamic therapy. Nano-Micro Lett.

[235] Dong Z, Feng L, Chao Y, Hao Y, Chen M, Gong F (2019). Amplification of tumor oxidative stresses with liposomal fenton catalyst and glutathione inhibitor for enhanced cancer chemotherapy and radiotherapy. Nano Lett.

[236] Hu H, Deng X, Song Q, Yang W, Zhang Y, Liu W (2021). Mitochondria-targeted accumulation of oxygen-irrelevant free radicals for enhanced synergistic low-temperature photothermal and thermodynamic therapy. J Nanobiotechnol.

[237] Liu C, Ruan C, Shi R, Jiang B-P, Ji S, Shen X-C (2019). A near infrared-modulated thermosensitive hydrogel for stabilization of indocyanine green and combinatorial anticancer phototherapy. Biomater Sci.

[238] Yin J, Han Q, Zhang J, Liu Y, Gan X, Xie K (2020). MXene-Based Hydrogels Endow Polyetheretherketone with Effective Osteogenicity and Combined Treatment of Osteosarcoma and Bacterial Infection. ACS Appl Mater Interfaces.

